# Does IFRS information on tax loss carryforwards and negative performance improve predictions of earnings and cash flows?

**DOI:** 10.1007/s11573-023-01147-7

**Published:** 2023-04-29

**Authors:** Sandra Dreher, Sebastian Eichfelder, Felix Noth

**Affiliations:** 1 Faculty of Business and Economics, Otto-von-Guericke-Universität Magdeburg, Universitätsplatz 2, 39106 Magdeburg, Germany; 2 Department of Financial Markets, Halle Institute for Economic Research, Kleine Märkerstrasse 8, 06108 Halle (Saale), Germany

**Keywords:** Performance forecast, Out-of-sample tests, Deferred tax assets, Tax loss carryforwards, M40, M41, C53

## Abstract

We analyze the usefulness of accounting information on tax loss carryforwards and negative performance to predict earnings and cash flows. We use hand-collected information on tax loss carryforwards and corresponding deferred taxes from the International Financial Reporting Standards tax footnotes for listed firms from Germany. Our out-of-sample tests show that considering accounting information on tax loss carryforwards does not enhance performance forecasts and typically even worsens predictions. The most likely explanation is model overfitting. Besides, common forecasting approaches that deal with negative performance are prone to prediction errors. We provide a simple empirical specification to account for that problem.

**Supplementary Information:**

The online version contains supplementary material available at 10.1007/s11573-023-01147-7.

## Introduction

Using hand-collected data from German IFRS accounts, we investigate whether accounting information regarding deferred taxes from tax loss carryforwards and negative firm performance can improve predictions of future firm performance. Research provides evidence of a significant association between financial reporting information on deferred tax assets and future firm performance (Herbohn et al. 2010; Flagmeier 2017) that increases the explanatory power or forecasting regressions (Dhaliwal et al. 2013). Evidence also suggests a higher persistence of positive than negative current performance (Hayn 1995; Li 2011).

As outlined by the statistical literature (e.g., Hagerty and Srinivasan 1991; Konishi and Kitagawa 2008; Shmueli 2010) testing in sample (explanation) and out of sample (prediction) are distinct concepts that can require different types of models.^1^ Since previous research relies exclusively on in-sample testing, we contribute to the literature by analyzing whether information on negative current firm performance and deferred taxes from tax loss carryforwards improves predictions out of sample. Previous research relies exclusively on in-sample testing. Sharing the motivation of some work in other areas of accounting research (e.g., Ciconte et al. 2016), we therefore concentrate on out-of-sample testing.

Our analysis is of interest for at least three reasons. First, as shown by Ohlson (1995, 2001), future firm performance—earnings and cash flows—is value relevant.^2^ Thus, it is critical for investors, creditors, and other stakeholders to know if specific accounting items can improve predictions. Second, correctly specified predictions of future cash flows and earnings are essential for practitioners (e.g., analysts), as well as for many research questions in the accounting and finance literature (e.g., to calculate the cost of capital; see Fama and French 2006; Hou et al. 2012). Third, accounting standards’ essential target is to provide useful information about a firm’s financial position and performance. The complexity of accounting for deferred taxes and respective costs has often been criticized (Weber 2009; Laux 2013). Thus, standard setters and authorities such as the Financial Accounting Standards Board and the International Accounting Standards Board should consider whether such items’ information content helps improve predictions.

Corresponding to International Accounting Standard (IAS) 12.34, deferred tax assets from tax loss carryforwards are only recognized to the extent that the tax benefit’s realization is likely. This is only the case if the firms generate sufficient future (taxable) profits to offset tax loss carryforwards. It has been argued that firms can use additional deferred tax assets from tax loss carryforwards to signal positive future firm performance (Herbohn et al. 2010; Flagmeier 2017). Considering loss persistence, several studies argue that negative firm performance is typically less persistent (Hayn 1995; Li 2011). Transitory losses are a primary reason for that (Joos and Plesko 2005). Due to business cycles, economic shocks, restructurings, and similar issues, firms might be able to transform their current losses into future profits. Thus, positive performance outcomes are more persistent and have higher predictive validity than losses.

We use a unique hand-collected panel of firms listed on the German stock market that encompasses detailed information on deferred taxes and tax loss carryforwards from the tax footnote under International Financial Reporting Standards (IFRS). Unlike US Generally Accepted Accounting Principles (GAAP), the tax footnote of IFRS accounts contains mandatory details on the amount of unrecognized (i.e., the nonvaluable component of) tax loss carryforwards. Since this information should be based on a firm’s *internal* estimate of future taxable earnings, it could be a helpful predictor of future pre-tax earnings, post-tax earnings, and cash flows. In addition, we analyze the usefulness of voluntarily disclosed accounting information on tax loss carryforwards (i.e., the total amount of tax loss carryforwards, the book value, and changes in valuation allowances for deferred tax assets from tax loss carryforwards that are not mandatory under IFRS) for performance predictions. We confirm previous findings suggesting a negative association between unrecognized (deferred taxes from) tax loss carryforwards and future firm performance in in-sample tests (Herbohn et al. 2010; Flagmeier 2017). However, our out-of-sample tests, including a battery of robustness checks, reveal that such items typically reduce predictive validity.

A theoretical explanation for our finding is model overfitting (Shmueli 2010; Sarstedt and Danks 2022), which means that a model overfits its training data and thus also captures unstable relations. This problem is especially relevant for noisy predictors with potential measurement error. Regarding unrecognized tax loss carryforwards (ULCFs), there are three main reasons for measurement error. First, ULCFs result from the internal estimates of managers that by themselves can be subject to forecasting error. Lev et al. (2010) provide evidence that such estimate-based items are less useful for forecasting. Second, several papers suggest that ULCFs are used for earnings management (e.g., Schrand and Wong 2003; Gordon and Joos 2004), which reduces the accuracy of that information. Third, differences in tax and financial accounting induce additional measurement error if ULCFs are used for the prediction of consolidated earnings and cash flows.^3^

We also find robust evidence that common forecasting approaches that treat positive and negative performance similarly (e.g., Barth et al. 2001; Kim and Kross 2005; Dichev and Tang 2009; Lev et al. 2010) overestimate the persistence of current negative firm performance. This holds especially for long-run prediction horizons, increasing the likelihood of loss reversal. Considering differences in the persistence of negative and positive current performance in regressions significantly increases the explanatory power and predictive validity. In additional out-of-sample tests, we find mixed evidence for standard proxies of persistent and transitory losses (see also Joos and Plesko 2005; Li 2011).

We contribute to the literature in three ways. First, while in-sample tests typically find a significant association between deferred taxes from the tax loss carryforwards with future tax payments (Laux 2013; Flagmeier 2022) and firm performance (Flagmeier 2017, 2022; Herbohn et al. 2010), our out-of-sample tests show that such items typically worsen predictions. This holds even for after-tax cash flow predictions, suggesting the limited usefulness of deferred tax components in predicting cash taxes. Thus, our results should also be relevant to the literature on the information content of deferred taxes for future cash taxes.

Our outcome fits well with the results of Flagmeier (2022), who finds stock prices to be significantly associated with deferred tax items, but not with deferred tax assets from tax loss carryforwards. The combined results of Flagmeier’s and our study suggest that investors do not consider deferred tax assets from tax loss carryforwards in their investment decisions, since these items do not provide a robust signal of future firm performance. As the aim of accounting standards is not primarily to produce the best predictors for future firm performance, this does not mean that such information does not have relevant value. Nevertheless, in a cost–benefit analysis, it might also be interesting for standard setters to know which characteristics are relevant for performance predictions.

Second, we contribute to the literature on the information content of losses and negative cash flows that suggests a lower information content of losses than of profits (Hayn 1995; Joos and Plesko 2005; Li 2011). We find that considering the asymmetry in the information content of negative and positive performance with an interaction term of a dummy for negative performance and current performance enhances predictions. We find mixed evidence in testing the predictive validity of different proxy variables for persistent negative performance. Variables capturing the sequence of past losses and variables measuring the change in performance compared to the last year often significantly enhance (but also, in one specification, worsen) predictive validity. Other proxy variables (dividend-paying firms, firm–years with first-time negative performance) are inconclusive. While distinguishing between transitory and persistent negative performance is challenging, adding information on performance history can be useful for performance forecasts.

Finally, we contribute to the literature on forecasting and predictions. In line with Hagerty and Srinivasan (1991), Shmueli (2010), or Sarstedt and Danks (2022), we provide evidence that models can perform very differently in in-sample and out-of-sample testing. Thus, even the so-called true (i.e., correctly specified) explanatory model can underperform in predictions, since the minimization of expected prediction errors is statistically not equivalent to the minimization of the bias. In line with Shmueli (2010), we find limited predictive validity of accounting information on tax loss carryforwards that can by biased by (a) errors in the prediction of future performance, (b) earnings management, and (c) mismatches between tax and financial accounting. Our findings suggest that in-sample tests are not an appropriate statistic for identifying predictive validity and can lead to misleading results.

## Related literature and hypotheses

### Evidence

As mentioned before, evidence on the information content of deferred taxes from tax loss carryforwards relies on in-sample testing. Using UK GAAP data, Gordon and Joos (2004) find a significant and negative association between the sum of unrecognized deferred tax assets and pre-tax performance indicators. Legoria and Sellers (2005) and Jung and Pulliam (2006) use US GAAP data and find a significant association between future cash flows, respectively, cash flows and earnings, and changes in the valuation allowance on deferred tax assets. Christensen et al. (2008) find that observations with abnormally high valuation allowances in US GAAP annual reports have lower operating performance in future periods. Jackson (2015) finds a significant association between deferred taxes and future pre-tax earnings in US GAAP reports.

Only two papers explicitly consider information in financial reports on tax loss carryforwards. Herbohn et al. (2010) find a negative association between the valuation allowance regarding deferred tax assets from tax loss carryforwards and indicators of future firm performance (earnings, cash flows, and EBITDA) for Australian GAAP data. Flagmeier (2017) finds a negative and significant association between unrecognized tax losses (the same accounting measure as in our study) in IFRS accounts of German firms and future pre-tax cash flows and earnings. The author also finds a similar association between the US GAAP valuation allowance and future performance indicators. Some studies also find a significant association between accounting information on deferred taxes and future tax payments for US GAAP data (Laux 2013) and IFRS data (Flagmeier 2022).

We are aware of only one paper that tests the predictive validity of deferred tax items by out-of-sample tests. Chludek (2011a) uses the annual accounts of Standard & Poor’s 500 firms to test the explanatory power and predictive validity of deferred tax assets and deferred tax liabilities for future cash taxes. She finds that including deferred tax items increases explanatory power in sample but worsens out-of-sample predictions. Chludek’s analysis, however, does not allow for a statement of whether more detailed IFRS information on tax loss carryforwards might improve performance predictions, since it uses aggregate information of US GAAP accounts to predict cash taxes (and not firm performance).

Regarding the lower persistence of negative performance compared to positive performance, we are unaware of papers that analyze this heterogeneity’s effect on the quality of performance predictions by out-of-sample tests. While prediction models consider current performance as the main predictor, they typically do not account for differences in the persistence of negative and positive performance (e.g., Barth et al. 2001; Kim and Kross 2005; Hou and Robinson 2006; Dichev and Tang 2009; Lev et al. 2010; Bostwick et al. 2016). Exceptions are the works of Fama and French (2000) and Hou et al. (2012), who use a simple indicator variable for loss firms that only partially captures firms’ heterogeneity with positive and negative performance.

### Theoretical background and hypotheses

IAS 12.34 requires the recognition of deferred tax assets from the carryforward of tax losses and tax credits “to the extent that it is probable that future taxable profit will be available against that unused tax losses and unused tax credits can be utilized.” Under similar preconditions, IAS 12.24 requires the recognition of deferred tax assets from temporary deductible differences. Thus, the recognition is generally limited to the fraction of deferred tax assets expected to reduce future tax payments. By contrast, Accounting Standards Codification (ASC) 740 US GAAP requires a two-step approach. As a first step, deferred tax assets must be recognized for the total amount of unused tax losses, unused tax credits, and temporary deductible differences. In a second step, firms must reduce the amount of deferred tax assets by a valuation allowance (VAL) if the amount is more likely than not to be realized in the future (ASC 740-10-30-5).

While the net recognized amount is conceptually the same under both approaches, there are differences regarding the disclosure of the unusable amount and its composition. Under US GAAP, the VAL is a recognized contra-asset (i.e., non-valuable component) of deferred tax assets from unused tax losses, unused tax credits, and temporary deductible differences. Instead, IAS 12.81(e) requires a footnote disclosure of “the amount (and the expiry date if any) of temporary deductible differences, unused tax losses, and unused tax credits for which no deferred tax asset is recognized (…).” Thus, IAS 12 does not disclose the value of unrecognized tax assets from the sum of these components, but detailed information (the amount and potentially the expiry date) for each element.

There are two theoretical arguments why deferred tax assets in general and especially deferred tax assets from unused tax losses could be informative of future firm performance. First, deferred tax assets reflect future cash tax savings and should be instructive of future tax cash flows (Chludek 2011b; Laux 2013; Flagmeier 2022). If this is the case, they should also be informative of future after-tax cash flows.

Second and more relevant, the recognition in IAS 12 is only allowed to the extent that there are sufficient expected future profits against which the unused tax losses and unused tax credits can be utilized. Hence, IAS 12.34 requires managers to forecast future taxable income that can be offset against the remaining tax loss carryforwards. For example, if managers expect higher future earnings as well as higher taxable income, they might increase the amount of deferred tax assets from tax loss carryforwards and reduce the amount of ULCFs (Herbohn et al. 2010). Such an *internal* estimate can be a valuable predictor of future firm performance under the following requirements: (a) managers have better information than external stakeholders, (b) managers use the discretion provided by IAS 12.34 to signal future firm performance by a truthful estimate,^4^ and (c) future taxable income is strongly correlated with accounting measures of future firm performance (cash flows and earnings before and after taxes) and therefore helps to predict those items.

There are several reasons why these requirements might not be met. First, the internal estimates of managers can be subject to errors as well. Lev et al. (2010) provide evidence that estimate-based accounting items are less useful for performance predictions. Second, firms might manage their deferred tax assets from tax loss carryforwards for purposes such as a big bath or meeting analysts’ expectations. Several studies provide evidence that deferred tax positions and deferred tax assets from tax loss carryforwards are used for earnings management (e.g., Schrand and Wong 2003; Gordon and Joos 2004; Frank and Rego 2006; Christensen et al. 2008; Herbohn et al. 2010). Third, future taxable profits against which unused tax losses can be utilized might be a relatively poor predictor of future firm performance. Most relevant, taxable income differs in many ways from pre-tax earnings and cash flows (book–tax differences), depends on the jurisdiction, and is typically calculated at the single entity level and not on a consolidated level.^5^ Furthermore, specific tax provisions can limit the amount of taxable profits against which tax loss carryforwards can be utilized.

While most studies find that the unrecognized component of deferred tax assets from tax loss carryforwards is negatively associated with future firm performance in in-sample tests (e.g., Gordon and Joos 2004; Christensen et al. 2008; Herbohn et al. 2010; Jackson 2015; Flagmeier 2017), this does not necessarily mean that such items will help to improve predictions. Therefore, we empirically test the hypothesis that accounting information on unrecognized loss carryforwards enhances the prediction of future earnings and cash flows.

H1: *Considering accounting information on ULCFs improves the accuracy of performance predictions.*

In line with the literature, we do not use recognized deferred tax assets (from tax loss carryforwards) for our statistical tests but follow Flagmeier (2017), who uses unrecognized tax losses (ULCFs) for her analysis. The main reason for that approach is that deferred tax assets from tax loss carryforwards are positively affected by future taxable income and former tax losses.^6^ Following Flagmeier (2017) and Herbohn et al. (2010), we expect a negative association between ULCFs and future firm performance for two reasons: a) current losses predict negative future performance and b) *unrecognized* losses result from lower expectations of future (taxable) income as a proxy of future firm performance.

Our data show considerable heterogeneity of the tax footnote and voluntary disclosure behavior concerning tax loss carryforwards (see Sect. 3.1). For example, 28.9% of our firm*–*years *voluntarily* disclose the book value of a valuation allowance on deferred tax assets from tax loss carryforwards, and 51.7% the total amount of tax loss carryforwards.^7^ Considering the evidence (Gordon and Joos 2004; Herbohn et al. 2010; Dhaliwal et al. 2013) as well as the related literature on tax disclosure (e.g., Hamrouni et al. 2015; Flagmeier and Müller 2016; Lang 2017), it seems plausible to consider the (voluntary) disclosure of specific accounting information as a firm characteristic that could itself be useful for performance predictions. Hamrouni et al. (2015) and Jiao (2011) argue and provide evidence that firms with poor expected future performance choose a lower disclosure level and hide information from potential investors. Leung and Veenman (2018) find a positive association between the (voluntary) disclosure of non-GAAP earnings and future firm performance for loss firms. By contrast, the findings of Lang (2017) suggest that managers of firms with low expected performance choose a higher disclosure level to reduce analysts’ earnings expectations. Thus, future firm performance will be higher if firms voluntarily report non-GAAP earnings. Both mechanisms suggest that voluntary disclosure could be a helpful characteristic for predictions. We hypothesize the following.

H2: *Considering additional voluntarily disclosed accounting information on tax loss carryforwards improves the accuracy of performance predictions.*

Accounting research provides compelling evidence that losses are, on average, less persistent than profits (Hayn 1995; Joos and Plesko 2005; Li 2011). There are two main arguments for the lower persistence of negative firm performance. Hayn (1995) claims that the investors of a firm with negative performance can liquidate the firm (abandonment option). Investors will therefore limit their losses if they believe that losses are becoming persistent. In addition, some firms can turn current losses into future profits (Joos and Plesko 2005). Such transitory losses can be observed for growth-oriented firms (e.g., Amazon in the 2000s, Tesla in the 2010s), firms restructuring their business (e.g., Apple in the 1990s), or firms being hit by economic shocks (e.g., Lufthansa in the COVID-19 crisis). Thus, negative performance will be less persistent than positive performance, which suggests weaker associations for firm–years with negative performance (e.g., Joos and Plesko 2005; Li 2011).

Nevertheless, widely applied forecasting models of cash flows and earnings treat losses and negative cash flows equally with profits and positive cash flows (Barth et al. 2001; Kim and Kross 2005; Hou and Robinson 2006; Dichev and Tang 2009; Lev et al. 2010; Bostwick et al. 2016). Only a few papers explicitly account for the differences between firms with negative and positive current performance by adding a simple indicator variable for firms with losses (Fama and French 2006; Hou et al. 2012). Even that approach, however, will not be sufficient to capture the lower persistence of negative performance, since it does not account for differences in the association between current and future performance for firms with negative and positive current performance.

A simple example can document this. Assume that the average association between current performance and future performance is 1.1 for firms with positive performance (high persistence), 0.5 for firms with negative performance (low persistence), and 0.9 for the average of all firms (profit and loss firms). Thus, a profit of 100 in *t* would suggest a profit of 110 in *t* + 1, since profits are persistent and tend to grow. By contrast, a loss in *t* of – 100 would indicate a loss of – 50 in *t* + 1 as firms transform transitory losses into profits. Considering the average association of 0.9 would result in an underestimated profit for a profitable firm (90 in *t* + 1) and an overestimated loss for a loss firm (– 90 in *t* + 1). Since the size of the measurement error depends on the value of current performance, a simple loss dummy will not fully capture the heterogeneity in performance persistence. We hypothesize:

H3: *Considering the lower persistence of negative firm performance improves the accuracy of performance predictions.*

## Data and methodology

### Data

For our analysis, we use hand-collected accounting information from 2004 to 2012 from consolidated German annual reports from 2005 to 2012. In detail, we hand-collect the following information from the tax footnote: (a) ULCFs (mandatory item), (b) total tax loss carryforwards, and (c) a potential valuation allowance from tax loss carryforwards (all voluntary items under IFRS). We use this information to predict one- to four-year-ahead earnings and cash flows. We use Worldscope (Thomson Reuters 2012) to collect data on (future) firm performance from 2004 to 2015 and to complement our data with additional characteristics such as total assets.^8^

Consolidated IFRS accounts became obligatory for all listed firms on the German stock market in 2005.^9^ Since 2005 annual reports typically provide information on IFRS accounts from the preceding year, 2004, we consider that information as well. We manually collect consolidated IFRS accounts for all firms listed in the most relevant German stock indices (DAX 30 and MDAX 50) for at least one year, from 2005 until 2012. For example, suppose a firm was listed on the MDAX in 2005. In that case, we consider that firm’s data over the whole period to obtain a comprehensive time series of IFRS accounts. A total of 30 annual business reports were unavailable due to insolvencies, mergers, or acquisitions, even upon request via mail.^10^ For these firms, we consider all available business reports. Altogether, we collected 866 observations from 106 firms.

We adjusted observations with deviating fiscal years to the calendar year in which the fiscal year ended (e.g., 2012 for the fiscal year from October 2011 to August 2012). Loss carryforwards in foreign currencies were converted to euro values using the conversion rate on the accounting date. We excluded observations with incomplete fiscal years (12 observations), inconsistencies in the financial statements (three observations), or missing values (16 observations) regarding total assets, operating cash flows, and earnings before taxes at time *t* (*TA*_*t*_, *CFBT*_*t*_, *EBT*_*t*_) or cash flows and earnings at *t* + 1 (*CFBT*_*t*+*1*_, *EBTt*_+*1*_). Our final sample contains 835 observations of 106 firms. We provide detailed information on the sample composition in Table 1, Panel A.

**Table 1:** Sample information

Panel A: Sample composition										
	2004	2005	2006	2007	2008	2009	2010	2011	2012	Sum
Gross observations	73	92	97	103	104	100	100	99	98	866
Reduced by										
Incomplete fiscal year	1	4	2	1	1	1	0	1	1	12
Inconsistent statements	1	0	1	0	0	0	0	0	1	3
Missing values	2	0	1	1	3	0	1	0	8	16
Net observations (sample)	69	88	93	101	100	99	99	98	88	835

Panel B: Disclosure of information on losses and tax loss carryforwards											
Information	Mandatory	2004	2005	2006	2007	2008	2009	2010	2011	2012	Sum
Observations		69	88	93	101	100	99	99	98	88	835
Disclosure on											
DTA LCF	Yes	52	69	72	82	77	77	77	75	67	650
ULCF	Yes	35	51	65	71	74	75	75	75	66	586
TLCF	No	24	39	47	53	55	56	56	54	48	432
VAL	No	10	20	22	31	32	33	33	32	28	241
ΔVAL	No	11	16	12	9	17	16	15	13	12	121

The term *DTA LCF* denotes recognized deferred tax assets from tax loss carryforwards, *VAL* is the (voluntarily disclosed) book value of a valuation allowance on deferred tax assets from tax loss carryforwards, Δ*VAL* is the (voluntarily disclosed) change in a valuation allowance on deferred tax assets from tax loss carryforwards in year *t*, *ULCF* is the (mandatorily disclosed) unrecognized (i.e., the nonvaluable component of) tax loss carryforwards and *TLCF* is the (voluntarily disclosed) total tax loss carryforward

Our sample documents wide heterogeneity in disclosure behavior. While mandatory information on tax loss carryforwards is missing for a relevant fraction of firm–years, additional information is disclosed in many cases. Table 1, Panel B, provides detailed documentation of the disclosure behavior. As mandatory information, we consider recognized deferred tax assets from tax loss carryforwards, *DTA LCF*. The variable *ULCF* describes the amount of unused tax losses for which no deferred tax asset has been considered. We also report voluntary disclosures of the book value of valuation allowances regarding deferred tax assets from tax loss carryforwards, *VAL*. If voluntarily reported, we further document changes in the valuation allowance, Δ*VAL*, and the total amount of tax loss carryforwards *TLCF* (i.e., the sum of recognized tax loss carryforwards and ULCFs). If possible, we calculate Δ*VAL* from information on *VAL*.

Information on deferred tax assets from tax loss carryforwards is provided in the tax footnote in only 77.8% of the observations and on ULCFs in only 70.2%, despite these disclosures being mandatory according to IAS 12.81. In the case of deferred tax assets from tax loss carryforwards, this lack is mainly driven by observations disclosing an aggregate sum of all deferred tax assets (e.g., from tax loss carryforwards and timing differences) of different components of deferred tax assets (i.e., from tax loss carryforwards and interests) or the net of deferred tax assets and deferred tax liabilities. Similar issues hold for ULCFs.^11^ While part of the relatively high non-disclosure of mandatory items could be subject to the IFRS introduction period (Table 1, Panel B), our findings also raise doubt regarding the quality of IFRS accounting practices in German listed firms.^12^

As documented by the accounting literature (e.g., Becker et al. 2021; Ewert and Wagenhofer 2019; Tsalavoutas et al. 2020), low compliance levels with accounting regulations may be a consequence of low detection risks and required penalties. Indeed, the Wirecard scandal of 2020 (McCrum 2022) can be interpreted as a case study of weaknesses in the German public enforcement system and related auditing and corporate governance practices. Nevertheless, the noncompliance in our data could also indicate a lack of materiality of the non-reported information for the corresponding firms (Becker et al. 2021; Bischof et al. 2021; Tsalavoutas et al. 2020).

We observe a significant number of observations with additional voluntarily disclosed information: 28.9% report the book value of the valuation allowance on deferred tax assets from tax loss carryforwards, 14.5% the change of the corresponding valuation allowance in the current year, and more than half (51.7%) the aggregate amount of total tax loss carryforwards. Mandatory and voluntary disclosures increase moderately over time.

The statistics in Table 1, Panel B, have two important implications. First, although we want to address the predictive validity of mandatorily and voluntarily disclosed IFRS information, not all observations disclose that information. Thus, our empirical specification must account for the fact that not all observations provide the same information in the financial reports. To address this problem, we use indicator (i.e., dummy) variables to account for observations disclosing (*D* = 1) or not disclosing (*D* = 0) a particular type of information.

Second, disclosure behavior could be related to future firm performance. Hamrouni et al. (2015), Jiao (2011), and Leung and Veenman (2018) provide evidence that firms with poor performance will choose a lower disclosure level, and, therefore, voluntary disclosure signals higher future firm performance. By contrast, Lang’s (2017) findings suggest that managers of firms with bad news can also increase voluntary disclosure to create lower earnings expectations for analysts and investors. Therefore, from the perspective of an external person who wants to predict future firm performance (e.g., an analyst), the disclosure of certain accounting items could be a relevant firm characteristic for performance prediction. By including disclosure indicator variables, we control for differences in firms’ future performance with high and low disclosure levels.

We use the general industry classification of Thomson Reuters (2012) to allocate our observations to industries. By far the majority of our observations are industrial firms (617 firm–years). Other relevant industries are public utilities (23 firm–years), transportation (27 firm–years), banking (61 firm–years), insurance (43 firm–years), financial services, and others (73 firm–years). Due to the limited number of observations, we abstain from a more precise industry classification. We do not exclude financial firms or potential outliers from our primary setting. In an untabulated robustness test, we also excluded financial firms and outliers. The corresponding results confirm our findings.

### Methodology

Our analysis is primarily based on Lev et al. (2010) who analyze mean absolute forecast errors (MAFEs) in out-of-sample tests (Lev et al. 2010; Eng and Vichitsarawong 2017). In doing so, we first perform a regression using a standard prediction model that we enrich with additional accounting information on deferred tax assets and tax losses. In the second step, we use these regression results to predict performance indicators and calculate the forecast error as the difference between the observable “true” performance (e.g., true cash flow in 2012) and its predicted value (e.g., predicted cash flow prediction in 2012). Since the error can be positive and negative, we take its absolute value and then calculate the mean of the absolute forecasting error for each year. Thus, the MAFE over *n* observations can be described as $MAFE = \frac{{\sum {|Actual - Forecast|} }}{n}$. We then perform a *t*-test if the errors of alternative prediction models differ significantly from each other.

Following Lev et al. (2010), we also calculate Theil’s U as an alternative statistic. Theil’s U is the unweighted average of U-statistics over all predicted years, where $U = \sqrt {{{\sum {\left( {Actual - Forecast} \right)^{2} } } \mathord{\left/ {\vphantom {{\sum {\left( {Actual - Forecast} \right)^{2} } } {\sum {\left( {Actual} \right)^{2} } }}} \right. \kern-0pt} {\sum {\left( {Actual} \right)^{2} } }}}$. Theil’s U virtually provides a weighted average statistic of absolute forecast errors, with higher weights on larger errors. Since Theil’s U is an aggregate statistic, we cannot test for statistically significant differences in errors (as in MAFEs). As complementary test statistics, we also calculate with in-sample tests whether specific variables increase the explanatory power of our models (see Online Appendix B).

We consider two pre-tax performance measures as the dependent variables for our analyses: a) cash flow from operations before taxes (*CFBT*) and b) earnings before taxes (*EBT*). For cash flows, we adjust the after-tax operating cash flow, as provided by our hand-collected data or by Worldscope (Net Cash flow – Operating Activities Field 04860), with current taxes corresponding to IAS 12.15 to approximate the pre-tax operating cash flow. In additional analyses, we consider the after-tax performance indicators c) cash flow from operations after taxes (*CFAT*) and d) earnings after taxes (*EAT*).

Following the literature on predicting future cash flows and earnings (Finger 1994; Lorek and Willinger 1996; Dechow et al. 1998; Lev et al. 2010), we rely on a simple standard prediction model. Lev et al. (2010) suggest that a parsimonious model with current performance as the only explanatory variable is well suited to predict future firm performance. Similar to Herbohn et al. (2010) and Flagmeier (2017), our baseline model regresses a measure of future firm performance (*PERF*_*t*+*x*_) on the same current performance measure (*PERF*_*t*_) and indicator variables for industry and year fixed effects. As an alternative robustness check, we also use the widely-applied prediction model of Barth et al. (2001; see Table A3, Panels C and D, in Online Appendix C). Corresponding to the work of Flagmeier (2017), we scale performance measures by total assets and do not explicitly control for firm size (for tests including firm size, see Online Appendix D). Thus, our baseline model is

1 $$PERF_{it + x} = \alpha + \beta_{1} \times PERF_{it} + \gamma_{1} \times INDUSTRY_{i} + \gamma_{2} \times YEAR_{t} + u_{it} .$$

To analyze the relevance of additional accounting items, we test whether the inclusion of these items increases the baseline model’s predictive validity (out-of-sample tests). If we consider all other variables, we obtain the following extended model:

2 $$\begin{gathered} PERF_{it + x} = \alpha + \beta_{1} \times PERF_{it} + \beta_{2} \times NPI_{it} + \beta_{3} \times NPI_{it} \times PERF_{it} + \beta_{4} \times D_{it}^{ULCF} + \beta_{5} \times D_{it}^{ULCF} \times ULCF_{it}^{{}} \hfill \\ + \beta_{6} \times VD_{it}^{TLCF} + \beta_{7} \times VD_{it}^{TLCF} \times TLCF_{it}^{{}} + \beta_{8} \times VD_{it}^{VAL} + \beta_{9} \times VD_{it}^{VAL} \times VAL_{it}^{{}} \hfill \; \\ \quad \quad \quad \quad \quad \;\;\; + \beta_{10} \times VD_{it}^{\Delta \;VAL} + \beta_{11} \times VD_{it}^{\Delta \,VAL} \times \Delta \,VAL_{it}^{{}} + \beta_{12} \times D_{it}^{DTA\;LCF} + \beta_{13} \times D_{it}^{DTA\;LCF} \times DTA\;LCF_{it}^{{}} \\ \quad \quad \quad \quad \;\; + \beta_{14} \times D_{it}^{DTAD} \times DTAD_{it}^{{}} + \beta_{15} \times D_{it}^{DTL} \times DTL_{it}^{{}} + \gamma_{1} \times INDUSTRY_{i} + \gamma_{2} \times YEAR_{t} + u_{it} . \hfill \\ \end{gathered}$$

In this model, the negative performance indicator *NPI* is a dummy variable for firm–years with negative current performance (*PERF*). It conforms to the forecasting approach of Fama and French (2006) and Hou et al. (2012). The interaction term $NPI \times PERF$ accounts for the lower expected performance persistence if *PERF* is negative. Research (e.g., Hayn 1995) suggests that positive outcomes of current performance are more strongly associated with future performance, *PERF*_*t*+*x*_, than negative performance outcomes. Therefore, we expect a positive and significant coefficient for *PERF* and a negative and significant coefficient for $NPI \times PERF$. While the *PERF* coefficient $\beta_{1}$ captures the persistence of positive performance outcomes, the expectedly lower persistence of negative performance outcomes is captured by the sum of the coefficients $\beta_{1}$ and $\beta_{3}$.

We enrich the model by ULCFs scaled by total assets (*ULCF*).^13^ To account for the variation in mandatory disclosure, we include the indicator variable $D_{{}}^{ULCF}$, which equals one if ULCFs have been reported. This approach has two advantages. First, $D_{{}}^{ULCF}$ controls for differences between observations reporting and not reporting that item. Therefore, it allows us to keep observations that do not report *ULCF*, which increases our sample size and reduces concerns of a non-representative sample and external validity. Second, $D_{{}}^{ULCF}$ enables us to test how a firm’s higher mandatory disclosure level is related to future firm performance. Thus, while $D_{{}}^{ULCF}$ accounts for the disclosure of ULCF as such, $D_{{}}^{ULCF} \times ULCF$ identifies the association of current ULCFs with future firm performance. The coefficient for $D_{{}}^{ULCF} \times ULCF$ captures the effect of the reported value of *ULCF* on future firm performance.^14^

A potential concern regarding our approach is (endogenous) sample selection, which is accounted for by models such as the Heckman model (e.g., Lennox et al. 2012). While such models are used to identify (typically causal) relations between variables, they are neither used in nor appropriate for performance predictions.^15^ In our analysis, we take the role of an external stakeholder who wants to predict future firm performance. From this perspective, all current observable characteristics are exogenous in a way that will not be affected by future performance outcomes (time lag) and can also not be affected by the external observer. Thus, while the accounting choice can be regarded as endogenous “self-selection” at the level of the firm, it is an exogenous characteristic for an outsider who intends to predict future firm performance.^16^

We further include a comprehensive set of indicator variables and interaction terms to test H2, suggesting a positive effect of voluntarily disclosed information on predictive validity. We consider the voluntary disclosure of a) total tax loss carryforwards (an indicator variable with a value of one if the information is disclosed, $VD_{{}}^{TLCF}$, with an interaction term with the disclosed value, $VD_{{}}^{TLCF} \times TLCF$); b) the book value of the valuation allowance on deferred tax assets from tax loss carryforwards (with the indicator variable $VD_{{}}^{VAL}$ and the interaction term $VD_{{}}^{VAL} \times VAL$); and c) the annual change in the corresponding valuation allowance (with the indicator variable $VD_{{}}^{\Delta \;VAL}$ and interaction term $VD_{{}}^{\Delta \;VAL} \times \Delta \,VAL$), with all accounting items scaled by total assets.

As in the related literature (Gordon and Joos 2004; Herbohn et al. 2010), we also consider accounting items on deferred taxes, including the (mandatory) disclosure of recognized deferred tax assets from loss carryforwards, $D_{{}}^{DTA\;LCF}$, and the corresponding interaction term $D_{{}}^{DTA\;LCF} \times DTA\;LCF$. Regarding deferred tax assets from timing differences, *DTAD*, and deferred tax liabilities, *DTL*, we abstain from including additional indicator variables and confine ourselves to the interaction terms $D_{{}}^{DTAD} \times DTAD$ and $D_{{}}^{DTL} \times DTL$. The variable $D_{{}}^{DTAD}$ is almost entirely collinear with $D_{{}}^{DTA\;LCF}$ and therefore does not provide additional information. The variable $D_{{}}^{DTL}$ has a value of one in 98.7% of our observations. There are only 11 observations with $D_{{}}^{DTL} = 0$, a number that seems too small for meaningful inferences. We also test models that include these indicator variables in untabulated robustness checks but find no relevant changes in results. We provide detailed variable definitions as Appendix in Table 8.

### Descriptive analyses

Table 2 reports the descriptive statistics of the most relevant variables of our sample. We report information on the full sample for firms disclosing information on ULCFs (Panel B) and for firms not disclosing information on ULCFs (Panel C). We present total assets in millions of euros and scale other variables by total assets. An exception is the market-to-book value *MTB*, which is scaled by the book value of equity. Average (median) total assets amount to €62.9 billion (€4.8 billion) in the full sample, €77.3 billion (€4.7 billion) in the ULCF sample, and €29.0 billion (€5.2 billion) in the non-ULCF sample. Thus, firms reporting ULCFs are larger. Average cash flow from operations to total assets, *CFBT* (earnings before taxes *EBT*), amounts to 9.1% (5.5%) in the full sample. About 9.9% (13.1%) of the observations report negative cash flows (earnings), as denoted by *NCFBT* (*NEBT*). Comparing Panels B and C reveals that observations disclosing ULCF report higher performance (earnings, cash flows, EBITDA) and smaller losses.

**Table 2:** Descriptive statistics

Variable	Observations	Mean	Median	S.D	Min	Max
Panel A: Baseline sample						
Total assets (millions €)	835	62,896	4,7845	206,817	132	2,202,423
CFBT	835	0.091	0.081	0.093	– 0.332	0.635
EBT	835	0.055	0.044	0.090	– 0.695	0.553
NCFBT	835	0.099	0.000	0.299	0.000	1.000
NEBT	835	0.132	0.000	0.338	0.000	1.000
NCFBT × CFBT	835	– 0.004	0.000	0.019	– 0.332	0.000
NEBT × EBT	835	– 0.008	0.000	0.041	– 0.695	0.000
DIV	835	0.021	0.010	0.101	0.000	2.744
EBITDA	821	0.104	0.099	0.093	– 0.575	0.585
SALES	816	0.923	0.848	0.650	0.020	5.307
MTB	784	2.278	1.717	3.305	– 7.425	76.512
RD	495	0.028	0.024	0.027	0.000	0.208
ULCF	586	0.073	0.018	0.177	0.000	2.050
DTA LCF	650	0.009	0.003	0.013	0.000	0.086
DTAD	648	0.029	0.024	0.024	0.000	0.139
DTL	824	0.043	0.037	0.033	0.000	0.384
TLCF	432	0.112	0.044	0.235	0.000	2.140
VAL	241	0.015	0.004	0.034	0.000	0.234
ΔVAL	121	0.002	0.000	0.004	– 0.003	0.033
Panel B: Sample reporting information on ULCFs (ULCF sample)						
Total assets (millions €)	586	77,304	4680	243,230	142	2,202,423
CFBT	586	0.087	0.077	0.093	– 0.332	0.635
EBT	586	0.049	0.041	0.088	– 0.695	0.499
NCFBT	586	0.108	0.000	0.310	0.000	1.000
NEBT	586	0.142	0.000	0.349	0.000	1.000
NCFBT × CFBT	586	– 0.004	0.000	0.021	– 0.332	0.000
NEBT × EBT	586	– 0.009	0.000	0.046	– 0.695	0.000
DIV	586	0.022	0.010	0.117	0.000	2.744
EBITDA	573	0.097	0.096	0.092	– 0.575	0.529
SALES	567	0.910	0.844	0.668	0.025	5.307
MTB	541	2.169	1.647	2.075	– 7.425	27.136
RD	312	0.029	0.022	0.030	0.000	0.208
ULCF	586	0.073	0.018	0.177	0.000	2.050
DTA LCF	500	0.009	0.003	0.013	0.000	0.086
DTAD	499	0.0289	0.023	0.023	0.000	0.125
DTL	580	0.0414	0.035	0.032	0.000	0.384
TLCF	315	0.0957	0.040	0.210	0.000	2.050
VAL	154	0.0168	0.003	0.040	0.000	0.234
ΔVAL	97	0.0013	0.000	0.004	– 0.002	0.033
Panel C: Sample not reporting information on ULCFs (non-ULCF sample)						
Total assets (millions €)	249	28,988	5275	51,425	147	228,630
CFBT	249	0.101	0.090	0.092	– 0.120	0.582
EBT	249	0.069	0.052	0.094	– 0.393	0.553
NCFBT	249	0.080	0.000	0.272	0.000	1.000
NEBT	249	0.108	0.000	0.312	0.000	1.000
NCFBT × CFBT	249	– 0.002	0.000	0.011	– 0.120	0.000
NEBT × EBT	249	– 0.005	0.000	0.292	– 0.393	0.000
DIV	249	0.019	0.011	0.041	0.000	0.430
EBITDA	248	0.118	0.111	0.097	– 0.312	0.585
SALES	249	0.952	0.860	0.607	0.020	3.049
MTB	243	2.518	1.817	5.066	0.210	76.512
RD	183	0.027	0.027	0.0199	0.000	0.107
DTA LCF	150	0.009	0.004	0.013	0.000	0.074
DTAD	149	0.031	0.028	0.027	0.000	0.139
DTL	244	0.048	0.041	0.036	0.000	0.201
TLCF	117	0.156	0.059	0.288	0.003	2.140
VAL	87	0.013	0.005	0.018	0.000	0.095
ΔVAL	24	0.002	0.001	0.004	– 0.003	0.013

Descriptive statistics of the full sample and the restricted ULCF sample reporting information on ULCFs. The variable definitions are provided in Table 8 in the Appendix

On average, the firms in our sample distribute 2.1% of their total asset values as dividends, which is a high value and about 38.3% of their pre-tax earnings. Non-ULCF firms distribute slightly less (1.9%) than ULCF firms (2.2%). Average sales amount to 92.3% of total assets and do not vary significantly between the different subsamples. Research and development (R&D) expenses, RD (2.8% of total assets in the full sample), are slightly higher in the ULCF sample (2.9% versus 2.7%). In comparison, EBITDA (10.4% of total assets in the full sample) and market-to-book values (227.8% of the book value of equity in the full sample) are more minor in the ULCF sample (9.7% versus 11.8% and 216.9% versus 251.8%, respectively).

Considering information on deferred tax assets, ULCFs are, on average, 7.3% of total assets. This is a relevant fraction. Recognized deferred tax assets from tax loss carryforwards comprise, on average, 0.9% of total assets. Deferred tax assets from timing differences, *DTAD*, and deferred tax liabilities, *DTL*, are both more relevant, at 2.9% and 3.5% of total assets, respectively. These values are very similar in the ULCF and non-ULCF samples.

Regarding voluntary disclosure, the most relevant item is the total sum of tax loss carryforwards, with a relatively high number of observations and an average value of 11.2% of total assets. By contrast, the fraction of (changes of) the corresponding valuation allowance to total assets is relatively small (1.5 for *VAL* and only 0.2% for Δ*VAL*). Comparing the ULCF and non-ULCF samples reveals that observations not disclosing ULCFs have higher total tax loss carryforwards *TLCF*, a higher median (but not mean) of the valuation allowance *VAL,* and more remarkable changes in the valuation allowance Δ*VAL*.

To conclude our comparison of firms that disclose and do not disclose ULCFs in their financial reports, we find several interesting differences. First, non-disclosing firms are smaller, on average. This fits well with the finding of the literature that the compliance costs of taxes and bookkeeping have large economies of scale (see the review of Eichfelder and Vaillancourt 2014). Therefore, smaller firms have a stronger incentive to reduce compliance costs by simplifying financial reporting. Second, firms that disclose ULCFs report lower earnings and cash flows and have a higher probability of losses. This is consistent with the consideration that firms tend to disclose ULCFs if these become materially more relevant (Becker et al. 2021; Bischof et al. 2021; Tsalavoutas et al. 2020). Third, the differences in other observed indicators (sales, R&D expenses, dividend payments, and market-to-book ratios) among these groups are relatively small. Overall, our findings do not suggest that firms that do not disclose ULCFs have the intention to hide information, and non-disclosure does not seem to be a red flag for low-quality firms.

We also compare our descriptive information with two studies using hand-collected information on deferred taxes from tax loss carry forwards. In a recent study on the impact of tax loss carryforwards on cash holdings, Heitzman and Lester (2022) rely on 6,884 observations of listed US firms from 2010 to 2015. Benefits from loss carry forwards in their data amount to 3.1% of total assets *TA*) and are therefore considerably higher than deferred tax assets from loss carryforwards in our sample (0.9%). At least in part, this is due to a difference in accounting systems. While net operating loss benefits in US GAAP are recognized *before deducting* the valuation allowance, the *DTA LCF* value in our data has already been corrected by ULCFs. If we weight ULCFs with a tax rate of 35%, we would obtain a slightly higher value for gross (i.e., uncorrected) benefits from loss carryforwards (3.5%), which is similar to the result of Heitzman and Lester (2022). The firms of Heitzman and Lester (2022) are significantly smaller (with an average TA value of $8.3 billion), have a slightly lower EBITDA (9.3% of TA) and market-to-book ratio (198.9%), and pay out fewer dividends (only 55.2% of observations pay dividends, compared to 80.1% in our sample) but have significantly higher R&D expenses (4.3%) than the firms in our sample.

In a study on the association between information on tax loss carryforwards and future firm performance, Flagmeier (2017) uses a hand-collected sample of 664 observations of German firms from 2010 to 2012. The author reports similar deferred taxes from tax loss carryforwards (0.9%, compared to 0.9% in our sample) and smaller ULCFs (6.1%, compared to 7.3% in our sample), average earnings (4.3%, compared to 5.5% in our sample), and cash flows (6.8%, compared to 9.1% in our sample).

## Analysis and results

### Multivariate associations of variables

We initially analyze the signs and significance levels of our explanatory variables’ regression coefficients in Eq. (2). In doing so, we estimate a restricted regression model including exclusively the most relevant variables *NPI,* $NPI \times PERF,$ $D_{{}}^{ULCF}$, and $D_{{}}^{ULCF} \times ULCF$, as well as the full model including all the additional explanatory variables of Eq. (2). In Online Appendix A, we also provide information on the bivariate correlations of the variables. We perform ordinary least squares (OLS) regressions in Table 3 for (a) one-year-ahead and three-year-ahead performances,^17^ with (b) earnings before taxes, *EBT*, and (pre-tax) cash flow from operations, *CFBT*, as the performance measures. We use robust standard errors clustered at the firm level to account for heteroscedasticity and autocorrelation (Petersen 2009). The variance inflation factors never exceed 3.04, and their average values range from 1.69 to 1.83. Thus, multicollinearity is not a problem. We report the regular* R*^*2*^ as well as the adjusted *R*^*2*^ values.

**Table 3:** Regression results

Model	1	2	3	4	5	6	7	8
Performance measure	Cash flow				Earnings before taxes			
Dependent variable	CFBT_t+1_	CFBT_t+3_	CFBT_t+1_	CFBT_t+3_	EBT_t+1_	EBT_t+3_	EBT_t+1_	EBT_t+3_
PERF	0.745*** (0.057)	0.564*** (0.071)	0.732*** (0.057)	0.570*** (0.071)	0.847*** (0.063)	0.592*** (0.145)	0.832*** (0.066)	0.577*** (0.144)
NPI	0.013 (0.010)	0.010 (0.019)	0.013 (0.010)	0.009 (0.020)	0.010 (0.010)	– 0.006 (0.016)	0.010 (0.010)	– 0.008 (0.017)
NPI × PERF	– 0.436*** (0.166)	– 0.422* (0.228)	– 0.442*** (0.164)	– 0.448* (0.252)	– 0.365*** (0.109)	– 0.625*** (0.173)	– 0.378*** (0.124)	– 0.659*** (0.182)
D^ULCF^	– 0.002 (0.004)	– 0.001 (0.006)	– 0.006 (0.004)	0.000 (0.007)	0.007 (0.005)	0.009 (0.008)	0.004 (0.005)	0.004 (0.009)
D^ULCF^ × ULCF	– 0.067*** (0.022)	– 0.034 (0.021)	– 0.062** (0.028)	– 0.059** (0.027)	– 0.051** (0.023)	– 0.107*** (0.039)	– 0.042** (0.021)	– 0.095** (0.045)
VD^TLCF^			0.003 (0.004)	– 0.000 (0.007)			– 0.005 (0.005)	– 0.000 (0.008)
VD^TLCF^ × TLCF			– 0.011 (0.016)	0.021 (0.026)			– 0.023 (0.016)	– 0.037 (0.035)
VD^VAL^			– 0.007 (0.005)	– 0.006 (0.008)			0.003 (0.005)	– 0.002 (0.009)
VD^VAL^ × VAL			0.041 (0.142)	0.104 (0.125)			0.001 (0.079)	0.046 (0.209)
VD^ΔVAL^			– 0.003 (0.005)	– 0.004 (0.009)			– 0.010 (0.006)	– 0.009 (0.009)
VD^ΔVAL^ × ΔVAL			0.230 (0.562)	1.437* (0.834)			0.726 (1.235)	1.189** (0.552)
D^DTA LCF^			0.005 (0.006)	0.005 (0.009)			0.004 (0.007)	0.005 (0.011)
D^DTA LCF^ × DTA LCF			– 0.107 (0.219)	0.244 (0.307)			0.093 (0.182)	0.344 (0.411)
D^DTAD^ × DTAD			0.106 (0.122)	– 0.110 (0.167)			0.090 (0.098)	– 0.033 (0.183)
D^DTL^ × DTL			– 0.079 (0.076)	0.029 (0.117)			– 0.084 (0.059)	– 0.114 (0.115)
Industry effects	Yes	Yes	Yes	Yes	Yes	Yes	Yes	Yes
Year effects	Yes	Yes	Yes	Yes	Yes	Yes	Yes	Yes
Observations	835	792	835	792	835	792	835	792
R^2^	0.644	0.504	0.647	0.508	0.617	0.352	0.623	0.359
Adjusted R^2^	0.636	0.492	0.635	0.490	0.608	0.337	0.610	0.336

The dependent variables are the operating cash flow before taxes *CFBT* at time *t* + 1, *CFBT* at time *t* + 3, earnings before taxes *EBT* at time *t* + 1, or *EBT* at time *t* + 3 (in each case scaled by total assets). The estimates are calculated by OLS. Heteroscedasticity-robust standard errors are clustered at the firm level and documented in parentheses. The superscripts ***, **, and * indicate statistical significance at the 1%, 5%, and 10% levels, respectively. The term *PERF* is the current value of the dependent variable. Table 8 in the Appendix provides detailed variable definitions

As expected, we observe a positive and significant coefficient for *PERF,* but a negative and significant coefficient for $NPI \times PERF$. Model 1 (5) in Table 3 suggests that a current *CFBT* (*EBT*) value of 10 percentage points of total assets, ceteris paribus, predicts a future *CFBT* (*EBT*) value at *t* + 1 of 7.45 (8.47) percentage points of total assets. This suggests a persistence of positive performance of 74.5–84.7%. If the current *CFBT* (*EBT*) values are negative, the aggregate effect on future performance can be calculated by the sum of the coefficients on *PERF* and $NPI \times PERF$. Thus, a negative performance at time *t* of 10 percentage points predicts a negative performance at *t* + 1 of *CFBT* of only 3.09 percentage points of total assets (= 0.745–0.436) and of *EBT* of 4.82 percentage points (= 0.847—0.365). In line with the literature (e.g., Joos and Plesko 2005; Li 2011), we find a higher persistence of positive performance outcomes and a lower persistence of losses.

By contrast, the coefficient of *NPI* does not differ significantly from zero. This finding has two implications. First, including the dummy *NPI* is insufficient to account for the heterogeneity in the persistence of firms with negative and positive performance. Second, in a horse race, the interaction term $NPI \times PERF$ outperforms the simple indicator variable *NPI*. Thus, adding $NPI \times PERF$ might also be helpful for predictions.

Confirming the evidence (Herbohn et al. 2010; Flagmeier 2017), we find a negative and significant coefficient for the interaction term $D_{{}}^{ULCF} \times ULCF$ that measures the effect of the reported value of ULCFs. This indicates that firms with more unrecognized tax loss carryforwards (a higher non-valuable component of tax loss carryforwards) have lower expected performance in the future. Nevertheless, this does not mean that this information improves predictions. The extended models confirm our findings on the negative and significant coefficients of $NPI \times PERF$ and $D^{ULCF} \times ULCF$ (Models 3, 4, 7, and 8).

In these models, we find almost no significant association between the different indicator variables considering mandatory ($D_{{}}^{ULCF}$, $D_{{}}^{DTA\;\;LCF}$) or voluntary ($VD_{{}}^{TLCF}$, $VD_{{}}^{VAL}$, $VD_{{}}^{\Delta \;VAL}$) disclosure and future firm performance. This also holds for additional (untabulated) regressions, including only part of these disclosure dummies. For three-year-ahead cash flows and earnings, we obtain a positive and significant association with changes in the valuation allowance captured by $VD^{\Delta VAL} \times \Delta VAL$. This association should be treated with caution, since we find no significant associations with one-year-ahead performances. The coefficient in Model 4 is significant only at the 10% level.

### Out-of-sample tests on predictive validity

We next turn to our out-of-sample tests addressing the predictive validity of our forecasting models. Since H1–H3 hypothesize an increase in performance forecasts’ predictive validity, the out-of-sample tests are our primary test statistic. To use as much information in our database as possible, we follow Lev et al. (2010) and use a rolling prediction window that considers the information of all available previous periods for predicting a given year *t*. Note that we enrich our data with information on future firm performance from Worldscope. Hence, while the explanatory variables for our hand-collected data on tax loss carryforwards and deferred taxes are limited to 2004–2012, performance outcomes are also available for 2013–2015.

We explain our statistical tests with the following example.

1. For the prediction of one-year-ahead cash flows in the year 2012 (2013 cash flows), we perform a regression of cash flows in 2012 and earlier years on once-lagged explanatory variables in 2011 and earlier years. Thus, we do not only consider data from the preceding year, since we want to rely on statistically robust relations that do not mainly depend on year cycles. We account for year cycles by the use of year dummies.
2. We use the information from the forecasting regression to predict cash flows in 2013 by using data from 2012.
3. Comparing the predicted cash flow in 2013 with the actual cash flow in 2013, we calculate the MAFEs and Theil’s U-statistics for the reference model and the more specific models that include additional variables.
4. We compare the reference and alternative models’ MAFEs and Theil’s U-statistics. In doing so, we perform t-tests to determine whether additional explanatory variables significantly reduce the MAFEs.

**Fig. 1:**
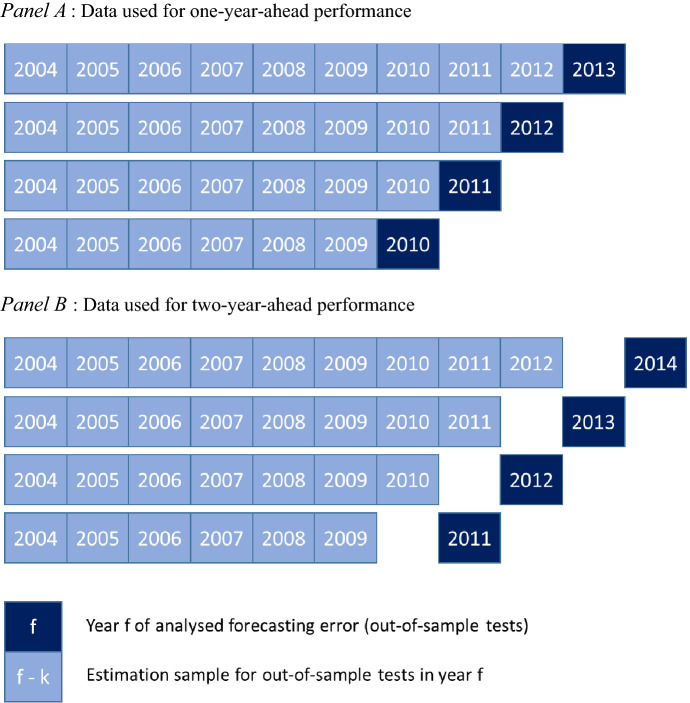
Data used for performance predictions

In the case of one-year-ahead (two-year-ahead) performance, we carry out this exercise for earnings and cash flows from 2010 to 2013 (2011–2014; see Fig. 1). As documented in Fig. 1, we generally use all earlier years’ information for our estimation sample. Predictions in earlier years rely on a smaller number of observations than predictions in later years.^18^ We regard more extended estimation periods and samples as beneficial, since correlations between variables can vary significantly between two years, due to macroeconomic shocks and they should revert to the mean in the long run. Thus, we use a minimum of six years for the regression sample, limiting the prediction sample to the years 2010–2013. We follow the same procedure for predictions of two-, three-, and four-year-ahead performance, but rely on explanatory variables lagged by more than one period.

We include industry and year indicator variables and industry and year fixed effects in all the models and perform two series of out-of-sample tests. In a *first series*, we test extended prediction models against the baseline model. The extended models generally add one category of additional variables to our baseline model from Eq. (1) (see also Table 4, Panel A). To test H1, Model ULCF adds the indicator variable *D*^*ULCF*^, denoting disclosure on *ULCF,* and the reported value of *ULCF* for firms reporting this information (*D*^*ULCF*^ × *ULCF*).

**Table 4:** Models for out-of-sample tests

Model	Definition
Panel A: Baseline model as the reference model	
Baseline	The model corresponds to Eq. (1), including current performance *PERF* and industry and year fixed effects. The model is used as a reference model for Models ULCF, VD, NPI, and NPERF
ULCF	Baseline model enriched by all variables on ULCFs: *D*^*ULCF*^ and *D*^*ULCF*^ × *ULCF*
VD	Baseline model enriched by all additional variables on voluntary disclosure regarding deferred taxes from tax loss carryforwards: *VD*^*TLCF*^, *VD*^*TLCF*^ × *TLCF*, *VD*^*VAL*^, *VD*^*VAL*^ × *VAL*, *VD*^Δ*VAL*^, and *VD*^Δ*VAL*^ × Δ*VAL*
NPI	Baseline model enriched by *NPI*
NPERF	Baseline model enriched by *NPI* and by the interaction term *NPI* × *PERF*
Panel B: Model NPERF as the reference model	
NPERF	Baseline model enriched by *NPI* and by the interaction term *NPI* × *PERF*. The model is used as a reference model for Models NPULCF, NPVD, and JOINT
NPULCF	Model NPERF enriched by all variables on ULCFs: *D*^*ULCF*^ and *D*^*ULCF*^ × *ULCF*
NPVD	Model NPERF enriched by all additional variables on voluntary disclosure regarding deferred taxes from tax loss carryforwards: *VD*^*TLCF*^*, VD*^*TLCF*^ × *TLCF, VD*^*VAL*^*, VD*^*VAL*^ × *VAL, VD*^Δ*VAL*^, and *VD*^Δ*VAL*^ × Δ*VAL*
JOINT	Model NPERF enriched by all additional explanatory variables in Eq. (2): *D*^*ULCF*^ and *D*^*ULCF*^ × *ULCF*, *VD*^*TLCF*^, *VD*^*TLCF*^ × *TLCF*, *VD*^*VAL*^, *VD*^*VAL*^ × *VAL*, *VD*^Δ*VAL*^, and *VD*^Δ*VAL*^ × Δ*VAL*, *D*^*DTA LCF*^, *D*^*DTA LCF*^ × *DTA LCF*, *VD*^*DTAD*^ × *DTAD*, and *D*^*DTL*^ × *DTL*
Panel C: LASSO models	
Adaptive LASSO	Starting with the JOINT model, we apply the adaptive LASSO method to obtain an optimal prediction model
Square-root LASSO	Starting with the JOINT model, we apply the square-root LASSO method to obtain an optimal prediction model

This table presents the documentation of the prediction models. We provide detailed variable definitions in Table 8 in the Appendix

To test H2, Model VD considers all the variables on the voluntary disclosure of total tax loss carryforwards *TLCF* and the valuation allowance *VAL* (specifically, *VD*^*TLCF*^, *VD*^*TLCF*^ × *TLCF*, *VD*^*VAL*^, *VD*^*VAL*^ × *VAL*, *VD*^Δ*VAL*^, and *VD*^Δ*VAL*^ × Δ*VAL*). Regarding Model VD, we also tested alternative versions, including only part of the voluntary disclosure information on deferred taxes from tax loss carryforwards (e.g., only voluntary disclosure on total tax loss carryforwards *TLCF*). Since corresponding tests do not lead to substantially different evidence, we abstain from reporting these results, which can be provided upon request.

To test H3, we consider two alternative models. Model NPI exclusively includes an indicator variable with a value of one in the case of negative current performance (*NPI*). Model NPERF further adds an interaction term of this variable and the (negative) performance of the firm (*NPI* × *NPERF*). Thus, unlike Model NPI, Model NPERF considers differences in earnings persistence between observations with positive and negative performance (see Table 3). We document these models in detail in Table 4, Panel A.

In a *second series*, we test whether adding information on deferred tax assets enhances predictions if the variables on performance persistence are already included. In these tests, we use Model NPERF (including *NPI* and *NPI* × *NPERF*) as a reference model and further add information on ULCFs (Model NPULCF) and the voluntary disclosure of information on deferred taxes from tax loss carryforwards (Model NPVD). Finally, we add a model including all variables from Eq. (2) and test that model against the Model NPERF. Thus, Model JOINT tests whether a combination of all variables might perform better than Model NPERF. We describe these models in detail in Table 4, Panel B.

**Fig. 2:**
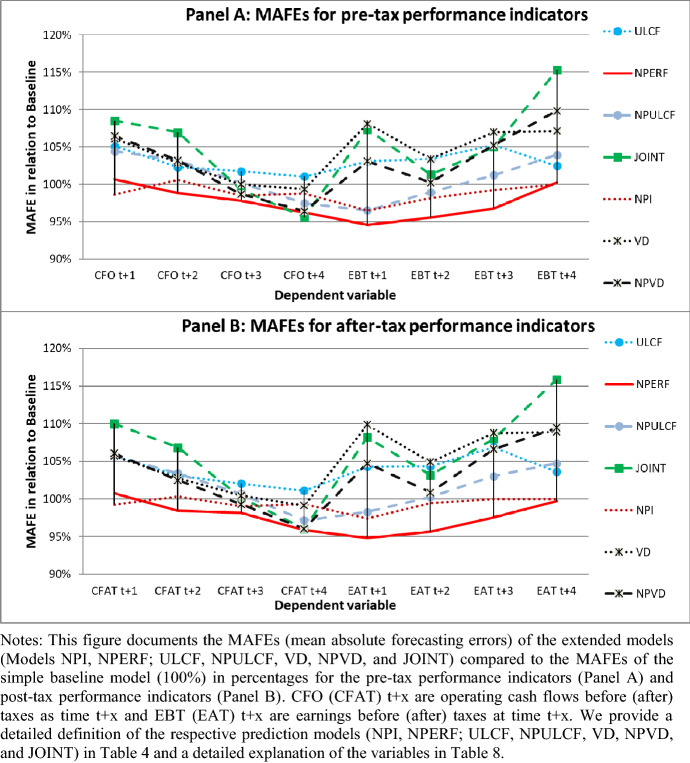
MAFEs as a percentage of the error of the baseline model

Initially, we provide graphical documentation of the MAFE statistics and the Theil’s U-statistics. Figure 2 documents the MAFEs of all the analyzed models (see Table 4) in percentages compared to the baseline model (100%). We report evidence on pre-tax performance in Panel A and on after-tax performance in Panel B. We see that forecasting errors of Model NPERF are, in almost all cases, lower than the baseline errors (< 100%), the NPI model errors, or the errors of any other models. By contrast, we find higher average errors for models that include information on tax loss carryforwards. Especially high errors can be observed for the JOINT, VD, NPVD, and ULCF models. An interesting observation is that the relative errors of the more comprehensive models (JOINT, NPULCF, NPVD, NPERF) decrease in the length of the prediction period (one to four years ahead) for cash flows but increase with the length of the prediction period for earnings.

**Fig. 3:**
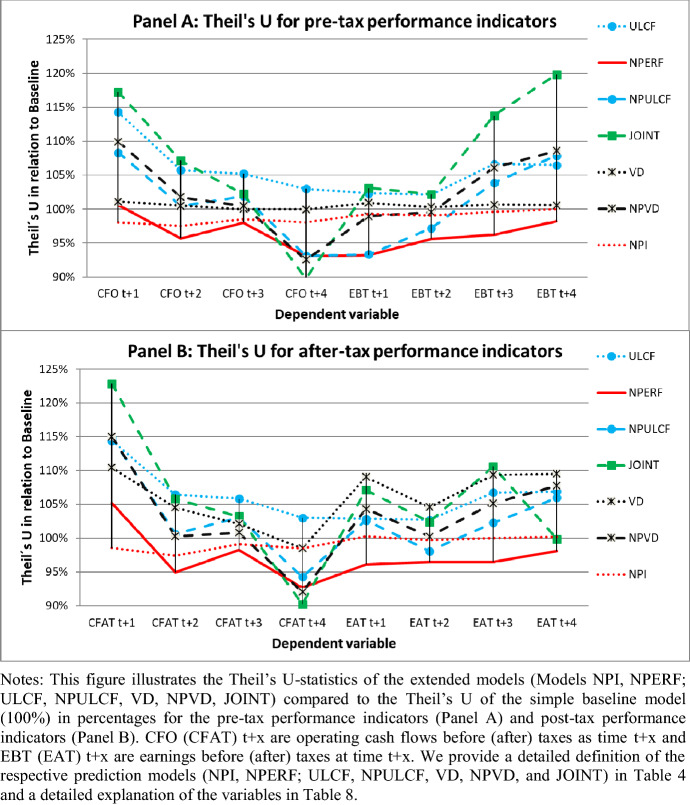
Theil’s U-statistics compared to Theil’s U of the Baseline model

Figure 3 documents the Theil U-statistics of all the analyzed models compared to the baseline model (100%). We can see that Model NPERF Theil’s U-statistics are, in almost all cases, lower than the baseline Theil’s U and that of Model NPI or of any other models. Exceptions are predictions of one-year-ahead cash flows (with a higher Theil’s U for Model NPERF) or four-year-ahead cash flows (with lower errors for Models NPULCF, NPVD, and JOINT). Instead, we observe higher Theil’s U-statistics for models including information on tax loss carryforwards. Exceptionally high Theil’s U-statistics can be observed for Model JOINT as well as for Models ULCF, VD, NPULCF, and NPVD. Similar to MAFEs, the relative Theil’s U-statistics of the more comprehensive models (Models JOINT, NPULCF, NPVD, and NPERF) decrease in the length of the prediction period (one to four years ahead) for cash flows but increase with the length of the prediction period for earnings.

We document the results of our out-of-sample tests in Table 5. Panel A documents the results of the out-of-sample test against the baseline model, while Panel B provides the test results against the NPERF Model. In both cases, we initially report the reference models’ (baseline or NPERF) *absolute values* of the MAFEs and of the Theil’s U-statistics (in parentheses). We multiply all values by 100 for readability.^19^

**Table 5:** Out-of-sample tests: Pre-tax performance

Model	1	2	3	4	5	6	7	8
Performance measure	Cash flow from operations before taxes				Earnings before taxes			
Prediction years	1 year (CFBT_t+1_)	2 years (CFBT_t+2_)	3 years (CFBT_t+3_)	4 years (CFBT_t+4_)	1 year (EBT_t+1_)	2 years (EBT_t+2_)	3 years (EBT_t+3_)	4 years (EBT_t+4_)
Panel A: Baseline model as the reference model								
Baseline absolute	2.93 (16.32)	3.45 (20.31)	4.58 (42.07)	4.76 (53.49)	2.58 (23.08)	3.82 (40.17)	4.00 (43.26)	4.06 (51.61)
ULCF difference	0.15** (2.33)	0.08* (1.17)	0.08 (2.19)	0.05 (1.59)	0.08*** (0.54)	0.13** (0.86)	0.21** (2.87)	0.10 (3.35)
VD difference	0.18*** (1.84)	0.10** (1.02)	0.00 (0.90)	– 0.03 ( – 0.66)	0.21*** (1.53)	0.13* (1.60)	0.28*** (4.00)	0.29** (4.50)
NPI difference	– 0.04 ( – 0.32)	0.02 ( – 0.50)	– 0.07** ( – 0.62)	– 0.06** ( – 1.05)	– 0.09*** ( – 0.17)	– 0.07*** ( – 0.38)	– 0.03*** ( – 0.18)	0.00 ( – 0.03)
NPERF difference	0.02 (0.10)	– 0.04^+++^ ( – 0.87)	– 0.10***^/+^ ( – 0.84)	– 0.18** ( – 3.71)	– 0.14***^/+^ ( – 1.57)	– 0.17***^/+^ ( – 1.75)	– 0.13***^/ ++^ ( – 1.64)	0.01 ( – 0.93)
Panel B: NPERF model as the reference model								
NPERF absolute	2.95 (17.35)	3.42 (19.40)	4.48^+^ (41.23)	4.58 (49.79)	2.43 (21.51)	3.64 (38.42)	3.87 (41.62)	4.06 (50.67)
NPULCF difference	0.11* (1.26)	0.15** (0.96)	0.11** (1.65)	0.06 (0.03)	0.05 (0.03)	0.13** (0.62)	0.18* (3.31)	0.15 (4.99)
NPVD difference	0.17*** (1.52)	0.15** (1.23)	0.04 (1.04)	0.01 ( – 0.28)	0.22*** (1.33)	0.18** (1.57)	0.34*** (4.28)	0.39*** (5.36)
JOINT difference	0.23*** (2.71)	0.28*** (2.32)	0.07 (1.77)	– 0.03 ( – 1.76)	0.33*** (2.29)	0.22** (2.63)	0.33** (7.59)	0.61*** (11.16)
Observations	285	271	255	167	285	271	255	167

Panel A shows the absolute MAFEs of the baseline model multiplied by 100, with the corresponding Theil’s U-statistics multiplied by 100 in parentheses, as well as the differences of the MAFEs, with the corresponding differences of Theil’s U-statistics in parentheses (both multiplied by 100), for Models ULCF, VD, NPI, and NPERF compared to the baseline model. Panel B shows the absolute MAFEs, with Theil’s U-statistics in parentheses (both multiplied by 100), of the NPERF model and the differences of the MAFEs, with the differences of the Theil’s U-statistics in parentheses (both multiplied by 100), for the Models NPULCF, NPVD, and JOINT compared to the NPERF model. The superscripts ***, **, and * (^+++^, ^++^, and ^+^) indicate statistically significant differences in MAFEs compared to the reference model (Model NPI) at the 1%, 5%, and 10% levels, respectively. Table 4 provides detailed documentation of the prediction models. We provide the variable definitions in Table 8 in the Appendix

For models tested against the reference model, we only report the *differences* between the MAFEs and the Theil’s U-statistics (again multiplied by 100 for readability) to simplify interpretation. Thus, we subtract the absolute value of the reference model from the tested model. For example, the value of the ULCF difference of 0.15 for the prediction of one-year-ahead cash flows (*CFBT*_*t*+*1*_) in Table 5, Panel A suggests that the MAFE increases by 0.15 (5.1% of the error of the baseline model of 2.93) if the additional variables of Model ULCF are added to the baseline model. In similar terms, the value of the NPERF difference for the prediction of two-year-ahead earnings (*EBT*_*t*+*2*_) suggests that the MAFE decreases by 0.17 (4.5% of the error of the baseline model of 3.82) if the additional variables of Model NPERF are added to the baseline model.

Note that the absolute values of the MAFEs and Theil’s U-statistics can be calculated by adding the reported differences to the reference model’s absolute values (the baseline model in Panel A and Model NPERF in Panel B). Thus, the absolute MAFE of Model ULCF in Panel A for predicting *CFBT*_*t*+*1*_ is 3.08 (= 2.93 + 0.15), while the absolute value of Theil’s U is 18.65 (= 16.32 + 2.33). In similar terms, the absolute MAFE of Model JOINT in Panel B for predicting *EBT*_*t*+*3*_ is 4.20 (= 3.87 + 0.33), while the absolute value of Theil’s U is 49.21 (= 41.62 + 7.59).

To identify statistically significant deviations, we perform one-sample t-tests on the equality of the MAFEs between the tested model and the reference model (either the baseline model or Model NPERF). For Model NPERF, we further investigate whether the MAFE is statistically significantly different from the MAFE of Model NPI. Therefore, we test whether adding information on the interaction term $NPI \times PERF$ significantly increases or reduces errors. We denote significant differences in the errors of Model NPERF against the baseline (Model NPI) by *, **, and *** (by ^+^, ^++^, and ^+++^).

Panel A of Table 5 documents that neither Model ULCF nor Model VD significantly reduce prediction errors in any specification. By contrast, we find that using both models significantly *increases* errors (documented in the *positive and significant differences* of MAFEs). For both models, we never see a negative and significant error difference. By contrast, we find increases (positive changes) in errors for Model ULCF in all specifications (for Model VD in seven of eight specifications). For the Model ULCF (Model VD), five (six) error changes differ significantly from zero. In addition, Theil’s U values indicate that Models ULCF and VD effectively increase errors.

On the other hand, Models NPI and NPERF typically reduce MAFEs and Theil’s U. We find a statistically significant reduction of errors for the prediction of three- and four-year-ahead cash flows, as well as for one- to three-year-ahead earnings. These error reductions range from 2.2% to 5.4% (0.8% to 3.5%) of the baseline model’s error for Model NPERF (Model NPI). Comparing the results of Models NPI and NPERF suggests that the latter outperforms the former.^20^ Overall, Table 5, Panel A, documents that Model NPERF outperforms the other models, while Models ULCF and VD increase prediction errors.

In Table 5, Panel B, we extend Model NPERF with additional information (e.g., on unrecognized loss carryforwards, *ULCF*) and test these augmented models against Model NPERF as a point of reference. Again, Model NPERF outperforms the other models. By contrast, Model ULCF significantly increases errors in five of eight specifications compared to Model NPERF. For Model NPVD, we find significant increases in the MAFEs in six of eight specifications, while Theil’s U increases in seven of eight specifications. We obtain almost identical results for the comprehensive Model JOINT, including all Eq. (2) variables. In addition, in relation to Model NPERF, Model JOINT significantly increases prediction errors for one- and two-year-ahead cash flows and all earnings specifications. These increases in errors are also relevant from a quantitative perspective. Compared to the absolute MAFE of Model NPERF, errors increase by 5.7% (*EBT*_*t*+*2*_) to 15.0% (*EBT*_*t*+*4*_). By contrast, for Model JOINT, we find no significant reduction in the MAFEs and increases in Theil’s U in seven of eight specifications.

The results of Table 5, Panel B, underline that adding information on tax loss carryforwards and corresponding deferred taxes to Model NPERF significantly reduces predictive validity. This finding is also interesting from a methodological perspective. As documented by in-sample tests in Table A.2 in Online Appendix B, models with a high number of variables (e.g., Model JOINT) typically increase explanatory power. Thus, the unexplained variation of the dependent variable *decreases*. However, this mechanism of reducing errors by adding more variables does not work for out-of-sample tests. The main reason for that is likely model overfitting, which results in unstable relations among variables that depend on the relevant test data set. Adding variables only enhances the predictive validity if the statistical relation between both variables is sufficiently valid (Lev et al. 2010).

### Out-of-sample tests on after-tax performance

In the following, we present out-of-sample tests on the after-tax performance measures of cash flow from operations after taxes (*CFAT*) and earnings after taxes (*EAT*). A theoretical argument for the more substantial explanatory power of information on deferred taxes in this context is that deferred tax items can provide information on future cash taxes (Laux 2013; Flagmeier 2022). Thus, one could expect information on ULCFs (Model ULCF) or deferred tax assets (Models VD and JOINT) to become more relevant for after-tax cash flows.

Apart from using after-tax performance measures, our specifications conform to those of Table 5. We report the results in Table 6, which confirm our previous findings. Similar to the pre-tax variables, we find that Model NPERF outperforms all the other prediction models. Including variables that account for negative performance (*NPI*) as well as for the degree of negative performance and the differences in the performance persistence of firms with negative and positive performance ${(}\text{NPI} \times \text{PERF})$ reduces the MAFEs compared to those in the baseline model (Panel A). By contrast, enriching Model NPERF by additional variables on tax loss carryforwards and deferred taxes increases the MAFEs (Panel B). Again, including all variables of Eq. (2) (Model JOINT) leads to significantly higher MAFEs in six of eight specifications.

**Table 6:** Out-of-sample tests: After-tax performance

Model	1	2	3	4	5	6	7	8
Performance measure	Cash flow from operations after taxes				Earnings after taxes			
Prediction years	1 year (CFAT_t+1_)	2 years (CFAT_t+2_)	3 years (CFAT_t+3_)	4 years (CFAT_t+4_)	1 year (EAT_t+1_)	2 years (EAT_t+2_)	3 years (EAT_t+3_)	4 years (EAT_t+4_)
Panel A: Baseline model as the reference model								
Baseline absolute	2.80 (22.05)	3.22 (25.01)	4.35 (52.03)	4.57 (65.08)	2.33 (30.62)	3.47 (55.48)	3.64 (59.96)	3.59 (66.38)
ULCF difference	0.16** (3.18)	0.010** (1.61)	0.09* (3.06)	0.05 (1.97)	0.10*** (0.88)	0.15*** (1.50)	0.25*** (4.04)	0.13 (4.58)
VD difference	0.16*** (2.30)	0.09** (1.13)	0.02 (1.10)	– 0.04 ( – 0.97)	0.23*** (2.77)	0.17** (2.54)	0.32*** (5.62)	0.32*** (6.32)
NPI difference	– 0.02 ( – 0.32)	0.01 ( – 0.65)	– 0.04 ( – 0.46)	– 0.03 ( – 0.98)	– 0.06*** (0.08)	– 0.02** ( – 0.15)	0.00 (0.01)	0.00 (0.11)
NPERF difference	0.02 (1.15)	– 0.05^+++^ ( – 1.26)	– 0.08**^/+^ ( – 0.92)	– 0.19**^/+^ ( – 4.77)	– 0.12^***/+++^ ( – 1.19)	– 0.15***/^+++^ ( – 1.95)	– 0.09***^/+++^ ( – 2.09)	– 0.01 ( – 1.27)
Panel B: NPERF model as the reference model								
NPERF absolute	2.82 (23.20)	3.17 (23.75)	4.26 (51.11)	4.38 (60.31)	2.21 (29.43)	3.32 (53.53)	3.55 (57.87)	3.58 (65.12)
NPULCF difference	0.14** (2.13)	0.16** (1.42)	0.11** (2.51)	0.06 (1.04)	0.08** (2.00)	0.16** (0.89)	0.20* (3.46)	0.18 (5.25)
NPVD difference	0.15** (2.16)	0.13** (1.33)	0.05* (1.35)	0.01 ( – 0.40)	0.23*** (2.52)	0.18** (2.08)	0.33*** (5.19)	0.35*** (6.41)
JOINT difference	0.26*** (3.89)	0.27*** (2.71)	0.09 (2.59)	0.01 ( – 1.58)	0.31*** (3.38)	0.26*** (3.28)	0.38*** (8.45)	0.58*** (1.18)
Observations	279	265	249	167	279	265	249	167

Panel A shows the absolute MAFEs of the baseline model multiplied by 100, with the corresponding Theil’s U-statistics multiplied by 100 in parentheses, as well as the differences of the MAFEs, with the corresponding differences of Theil’s U-statistics in parentheses (both multiplied by 100), for Models ULCF, VD, NPI, and NPERF compared to the baseline model. Panel B shows the absolute MAFEs, with Theil’s U-statistics in parentheses (both multiplied by 100), of the NPERF model and the differences of the MAFEs, with the differences of the Theil’s U-statistics in parentheses (both multiplied by 100), for the Models NPULCF, NPVD, and JOINT compared to the NPERF model. The superscripts ***, **, and * (^+++^, ^++^, and ^+^) indicate statistically significant differences in MAFEs compared to the reference model (Model NPI) at the 1%, 5%, and 10% levels, respectively. Table 4 provides detailed documentation of the prediction models. We provide the variable definitions in Table 8 in the Appendix

### Least absolute shrinkage and selection operator analyses

While our analysis is based on parsimonious prediction models similar to Lev et al. (2010), the literature has also developed models that optimize predictions in sample by selecting explanatory parameters. A common approach is the least absolute shrinkage and selection operator (LASSO) method. Freyberger et al. (2020) use this method to select characteristics that explain share prices and find that LASSO methods can reduce prediction errors. However, this is not necessarily the case, since LASSO estimators do not optimize out-of-sample predictions but are—similar to OLS—focused on optimizing in-sample tests and in-sample predictions. This is primarily a concern in our case. Our sample is relatively small, ULCFs might be subject to considerable measurement error and earnings management, and their relevance for explanatory power is limited (Shmueli 2010).

Unlike OLS, LASSO methods not only minimize the sum of squared residuals with a given set of variables and a specific functional form, but also select a set of variables and functional forms that optimize the prediction of a dependent variable in sample. We use two alternative specifications: (a) the adaptive LASSO (Freyberger et al. 2020) and (b) a non-parametric square-root LASSO (Belloni et al. 2014). As Freyberger et al. (2020), we use the Bayesian information criterion for variable selection. We start with all variables from Eq. (2), including year and industry dummies.

First, we test with the whole data set, which variables are selected by the applied LASSO models. For pre-tax performance, the models choose *PERF* in all specifications and the interaction term *NPI* × *PERF* in 15 of 16 specifications (four estimates with cash flows and four estimates with earnings for each LASSO-type model). By contrast, the interaction term *D*^*ULCF*^ × *ULCF* is only selected in eight of 16 specifications, and other indicators of Eq. (2) in only a few specifications.^21^ These analyses confirm that Model NPERF is the most preferred.

Second, we perform out-of-sample tests of whether the applied LASSO estimation models enhance our predictions. Similar to Panel B of Table 5, we use Model NPERF as the reference. We report the absolute MAFEs and Theil’s U-statistics (both multiplied by 100) for this model, as well as the differences in the MAFEs and Theil’s U-statistics (multiplied by 100) of the adaptive LASSO and the square-root LASSO compared to Model NPERF. We perform out-of-sample tests in the same way as in Table 5 for pre-tax performance and report the results in Table 7. We find that LASSO methods are not necessarily sufficient to enhance predictive validity in out-of-sample testing. Especially for cash flows, we find significantly larger MAFEs compared to Model NPERF as a reference. For pre-tax earnings, the evidence is mixed, with lower errors for two-year-ahead earnings and higher errors for four-year-ahead earnings, as well as higher errors for one-year-ahead earnings for the square-root LASSO.

**Table 7:** LASSO method analyses

Model	1	2	3	4	5	6	7	8
Performance measure	Cash flow from operations				Earnings before taxes			
Prediction years	1 year (CFBT_t+1_)	2 years (CFBT_t+2_)	3 years (CFBT_t+3_)	4 years (CFBT_t+4_)	1 year (EBT_t+1_)	2 years (EBT_t+2_)	3 years (EBT_t+3_)	4 years (EBT_t+4_)
LASSO method analyses								
NPERF absolute	2.95 (17.35)	3.42 (19.44)	4.48^+^ (41.23)	4.58 (49.79)	2.43 (21.51)	3.64 (38.42)	3.87 (41.62)	4.06 (50.67)
Adaptive LASSO Difference	0.10** (0.84)	0.09 (1.13)	0.15* (2.42)	0.35*** (6.50)	0.07 ( – 0.23)	– 0.24** ( – 1.81)	– 0.04 (2.94)	0.27** (4.67)
Square-root LASSO Difference	0.12** (0.45)	0.05 (1.37)	0.14** (1.22)	0.37*** (6.20)	0.21*** (1.41)	– 0.21* ( – 0.90)	– 0.09 (2.92)	0.22* (3.94)
Observations	285	271	255	167	285	271	255	167

This table shows the absolute MAFEs multiplied by 100 of the NPERF model, with the corresponding Theil’s U-statistics multiplied by 100 in parentheses, as well as the differences of the MAFEs, with the corresponding differences of Theil’s U-statistics in parentheses (both multiplied by 100) of the adaptive LASSO and square-root LASSO models compared to the NPERF model. The superscripts ***, **, and * indicate statistically significant differences in MAFEs compared to the Model NPERF at the 1%, 5%, and 10% levels, respectively. We provide the variable definitions in Table 8 in the Appendix

The reason for the limited performance of the LASSO models is that in-sample and out-of-sample testing are conceptually different (Shmueli 2010).^22^ Thus, in line with the literature (e.g., Konishi and Kitagawa 2008; Shmueli 2010), our findings suggest that the optimization of predictive validity requires out-of-sample testing to realize an optimal outcome (e.g., Sarstedt and Danks 2022).

### Robustness checks

We perform several robustness checks that are documented in detail in Online Appendix C. As mentioned earlier, there might be a concern that self-selection regarding the (mandatory) disclosure of information on ULCFs could affect our results. Therefore, we show in a robustness check that the results of out-of-sample tests do not change significantly if we reduce our sample to observations that report ULCFs (Online Appendix C, Table A3, Panels A and B). Again, we find that Models ULCF and VD increase the MAFEs and Theil’s U if compared to the baseline model, while Model NPI and especially Model NPERF reduce the MAFEs. Thus, the best choice for predictions is Model NPERF.

In a second test, we consider the widely applied model of Barth et al. (2001, BCN hereafter) as an alternative reference model (see also Bostwick et al. 2016, with further references). This model regresses future cash flow on current cash flow and six accruals: the current year’s change in accounts receivable (Δ*AR*), the change in accounts payable (Δ*AP*), the change in inventories (Δ*INV*), depreciation (*DEPR*), amortization (*AMORT*), and other changes in accruals (*OTHER*), where *OTHER* is the difference in earnings before taxes and operating cash flow adjusted by the five other accrual items (*OTHER* = *EBT – CFO* + Δ*AR –* Δ*AP* + Δ*INV* + *DEPR* + *AMORT*). The results are documented in Online Appendix C, Table A3, Panels C and D, and confirm our main findings.^23^

In a third test (not tabulated), we exclude observations from the year 2004, since IFRS financial reporting was introduced in 2005, which can limit the validity of the information from 2004. While we generally find a lower number of significant differences in the MAFEs (lower robustness of a smaller data set), our findings remain qualitatively unchanged. In a fourth test (not tabulated), we adjust our data. We exclude financial firms’ observations (banks, insurance companies, and other financial firms) and outliers from our sample. The remaining sample comprises 614 observations of 85 firms, and the results remain broadly unchanged. In a fifth test (not tabulated), we use EBIT and EBITDA (both scaled by total assets) as alternative dependent variables with consistent results. In a sixth set of tests (not tabulated), we consider alternative empirical specifications of our regression models. Most relevantly, we analyze itemized prediction models, where we test the predictive validity of the itemized explanatory factors of Model JOINT (e.g., *DTAD*).

### Additional analyses

We further test whether complementing Model NPERF with other explanatory variables improves predictions (Online Appendix D). Joos and Plesko (2005) and Li (2011) develop models to identify persistent negative performance and transitory negative performance. Testing critical indicators of their models, we find that considering the sequence of past losses and negative cash flows typically enhances predictive validity. Only in one specification do we see weak evidence at the 10% level that including such a variable results in higher forecast errors. We obtain similar results for including a variable considering a relative change in performance (weaker performance compared to the last year). By contrast, we find no conclusive evidence for an indicator variable for dividend-paying firms and an indicator variable for firm–years with first-time negative performance in the current year. Our evidence suggests that such variables to not improve out-of-sample predictions.

We further test whether including information on R&D activities enhances predictive validity, since high-R&D firms’ negative performance can be transitory (Kothari et al. 2002; Darrough and Ye 2007; Ciftci and Darrough 2015). In contrast to our expectations, considering R&D information decreases predictive validity. Finally, we find that the market-to-book ratio has only minor relevance for predictive validity and typically worsens Theil’s U-statistics. By contrast, including firm size typically enhances predictive validity. We also test models that include interaction terms of explanatory variables and industry dummy variables or industry trends (Online Appendix E). Our evidence suggests that the interaction terms of explanatory variables (e.g., current performance) and industry dummy variables can be useful for the prediction of earnings but not for the prediction of cash flows. Interaction terms of explanatory variables and industry-specific trends clearly worsen predictions.

## Conclusion

We analyze whether accounting information on tax loss carryforwards and negative performance helps to predict future firm performance, using a hand-collected panel of German-listed firms. While we find a negative association of unrecognized tax loss carryforwards (ULCF) with future firm performance, out-of-sample tests show that considering such information typically even worsens predictions because of model overfitting (Shmueli 2010; Sarstedt and Danks 2022). By contrast, accounting for the different informational values of negative and positive performance enhances performance predictions.

A limitation of our paper is the external validity of our findings. We rely on a relatively small sample of annual IFRS accounts of the largest public German firms. The results are thereby not representative of small firms or firms using different accounting standards. We look forward to research about the information content of tax loss disclosures using other settings and samples and potentially considering other outcomes than cash flows and performance as in our study.

## Electronic supplementary material

Below is the link to the electronic supplementary material.

Supplementary file1 (DOC 514 KB)

## Data Availability

The data that support the findings of this study are available from the corresponding author upon request.

## Appendix

**Table 8:** Variable definitions

Variable	Definition
Dependent variables	
CFAT_t+x_	Cash from operations after taxes scaled by total assets at time *t* + *x*
CFBT_+x_	Cash from operations before taxes scaled by total assets at time *t* + *x*
EAT_t+x_	Earnings after taxes scaled by total assets at time *t* + *x*
EBT_t+x_	Earnings before taxes scaled by total assets at time *t* + *x*
PERF_t+x_	Firm performance (measured either by cash flows or by earnings) scaled by total assets at time *t* + *x*
Explanatory variables for current firm performance	
CFAT_t_	Cash from operations after taxes scaled by total assets at time *t*
CFBT_t_	Cash from operations before taxes scaled by total assets at time *t*
EAT_t_	Earnings after taxes scaled by total assets at time *t*
EBT_t_	Earnings before taxes scaled by total assets at time *t*
PERF_t_	Firm performance (measured either by cash flows or by earnings) scaled by total assets at time *t*
NCFAT	Indicator variable for observations with negative after-tax cash flows
NCFBT	Indicator variable for observations with negative pre-tax cash flows
NEAT	Indicator variable for observations with negative after-tax earnings
NEBT	Indicator variable for observations with negative pre-tax earnings
NPI	Indicator variable for negative firm performance (measured either by cash flows or by earnings)
NPI × PERF	This interaction term of NPI and PERF accounts for differences in association of future performance to current performance for firm–years with negative performance compared to firm–years with positive performance. Potential versions include NCFAT × CFAT, NCFBT × CFBT, NEAT × EAT, and NEBT × EBT
Explanatory variables for ULCFs	
D^ULCF^	Indicator variable with a value of one if the information on ULCF is disclosed
D^ULCF^ × ULCF	Interaction term of D^ULCF^ and ULCFs scaled by total assets
Explanatory variables for voluntary disclosure	
VD^TLCF^	Indicator variable for the disclosure of total tax loss carryforwards, TLCF
VD^TLCF^ × TLCF	Interaction term of VD^TLCF^ and total tax loss carryforwards scaled by total assets
VD^VAL^	Indicator variable for disclosing the book value of a valuation allowance on deferred tax assets from tax loss carryforwards, VAL
VD^VAL^ × VAL	Interaction term of VD^VAL^ and the book value of a valuation allowance on deferred tax assets from tax loss carryforwards scaled by total assets
VD^ΔVAL^	Indicator variable for disclosing the change in a valuation allowance on deferred tax assets from tax loss carryforwards, ΔVAL
VD^ΔVAL^ × ΔVAL	Interaction term of VD^ΔVAL^ and the change in a valuation allowance on deferred tax assets from tax loss carryforwards in the current year scaled by total assets
Additional variables for deferred taxes (from tax loss carryforwards)	
D^DTA LCF^	Indicator variable for the disclosure of deferred tax assets from tax loss carryforwards, DTA LCF
D^DTA LCF^ × DTA LCF	Interaction term of D^DTA LCF^ and DTA LCF scaled by total assets
D^DTAD^ × DTAD	Interaction term of an indicator variable for disclosing deferred tax assets from timing differences and tax credits (DTAD) and DTAD scaled by total assets
D^DTL^ × DTL	Interaction term of an indicator variable for the disclosure of deferred tax liabilities (DTL) and DTL scaled by total assets
Additional firm information (relevant for descriptive statistics)	
DIV	Dividend payment scaled by total assets
EBITDA	Earnings before interests, taxes, depreciastion and amortization scaled by total assets
SALES	Total sales scaled by total assets
MTB	Market to book ratio (market capitalization scaled by the book value of equity)
RD	Expenses for research and development scaled by total assets

## Footnotes

1 In-sample tests use a given data set to identify relations between variables, including the assumed causes and effects. Out-of-sample tests use subsamples of a data set (training data) to predict variables in other subsamples (testing data). Thus, the intention is not to identify (causal) relations among variables, but to minimize prediction errors. Research provides evidence that models that enhance explanatory power do not necessarily improve out-of-sample predictions (e.g., Sarstedt and Danks 2022). Out-of-sample tests contribute substantially to understanding the usefulness of accounting items, since they simulate what most investors and analysts do: ‘predict, with no ex-post information (as implicitly assumed by in-sample studies), future earnings and cash flows’ (Lev et al. 2010, p. 784).

2 The empirical literature also provides evidence that accounting standards affect the information content of stock prices (e.g., Wang and Yu 2015).

3 Deferred tax assets (from tax loss carryforwards) result from the projection of future taxable income at the entity level. In addition, restrictions in the settlement of tax losses with future taxable income could reduce the predictive ability of this item.

4 A reason for standard-conforming accounting is the (reputational) risk of financial restatements and unstable accounting practices, which can be substantial. Lys et al. (2015) denote standard-conforming accounting practices that unintentionally reveal managers’ private information through passive signaling.

5 For example, in the German corporate income tax code, only 5% of dividend income is typically regarded as taxable. In addition, taxable income is calculated for each legally distinct group entity (every single corporate unit) if no group taxation regime is applied, while earnings and cash flows in IFRS accounts will be consolidated at the group level.

6 For example, additional tax losses in the past increase the total amount of tax loss carryforwards, which can result in higher expected future tax savings but does not necessarily imply better future firm performance. Other papers also rely on the valuation allowance encompassing the value of unrecognized deferred tax assets (from tax loss carryforwards; see Herbohn et al. 2010; Dhaliwal et al. 2013). In this case, the unrecognized losses are multiplied by the applicable tax rate, which differ among countries. Thus, a benefit of ULCFs as an explanatory variable is that it is not affected by differences in the various tax rates of a multinational group, which could bias unrecognized deferred taxes from tax loss carryforwards as a measure of non-valuable tax losses. For example, a firm with a high average tax rate and a low fraction of valuable tax losses can have the same deferred tax asset from tax loss carryforwards as a firm with a low average tax rate and a high fraction of valuable tax losses.

7 We regard the total amount of tax loss carryforwards as voluntarily disclosed if it is disclosed either directly or if firms report the amount of both unrecognized and recognized tax loss carryforwards.

8 We only had data access until 2015 when we collected these additional data from Worldscope.

9 Firms listed at non-European Union (non-EU) stock exchanges and using non-EU financial reporting standards were permitted to delay their adoption of IFRS until 2007 (see also PwC 2015).

10 To further complement our data, we also sent requests by mail to all observations with missing information on (a) deferred tax assets from tax loss carryforwards, (b) the amount of unused tax loss carryforwards that had not been recognized as deferred tax assets, and (c) the aggregate amount of recognized tax loss carryforwards and ULCFs in their annual reports.

11 It is possible that some observations do not disclose information, since the value of their tax loss carryforwards is zero. Nevertheless, if the tax footnote information does not allow for identifying such cases, we treat that information as missing. Note that we also sent a mail request to observations with missing information. We treat all observations without detailed information on either deferred tax assets from tax loss carryforwards or ULCFs as non-disclosures if either (1a) the corresponding values are reported in an aggregate sum with other items or (1b) the corresponding items are not reported and (2) our mailed request regarding these items was not answered.

12 Indeed, the non-disclosure of recognized deferred tax assets from tax loss carryforwards, *DTA LCF*, remains relatively stable over time. In the first two years of our sample, 23.0% of firm–years did not report this item, while 23.7% did not report this item in the last two years. In the case of ULCFs, we observe a fraction of 24.2% not reporting this footnote item in the last two years.

13 While parts of the literature refer to changes in the amount of unrecognized deferred tax assets (from tax loss carryforwards; see Gordon and Joos 2004; Herbohn et al. 2010), this item is not mandatory in IFRS. Thus, in line with Flagmeier (2017), we rely on ULCFs. We consider the carrying amount, since the use of annual changes would result in the loss of one observation period and the carrying amount encompasses information on changes of ULCF in earlier periods, increasing the variation in our data and the explanatory power of our models.

14 There might be concern that the coefficient estimates of $D_{{}}^{ULCF} \times ULCF$ are biased, since the (endogenous) dummy variable $D_{{}}^{ULCF}$ effectively uses firms with a reported ULCF value of zero as a reference point for firms that do not report ULCFs. Thus, one might think that excluding those 249 observations could be useful. However, this would generate a serious problem for external validity, since a significant fraction of even the largest listed German firms does not report ULCFs. Hence, prediction models must also provide valid results for firms that do not report all obligatory items. Endogeneity is not a serious problem for our prediction models, since such models do not intend to identify causal relationships but simply to minimize prediction errors. We also show in Online Appendix E that our results remain almost unchanged if we choose another reference point for firms not reporting ULCFs (e.g., the sample mean or the sample median of the value of *ULCF*).

15 The reason is that the targets of prediction models differ from those of explanatory models. While explanatory models intend to identify causal relationships among variables, prediction models intend to reduce prediction errors (Hagerty and Srinivasan 1991; Shmueli 2010; Sarstedt and Danks 2022).

16 From this perspective, the question is rather which functional form of the information at the disclosure level might be considered (Jiao 2011; Hamrouni et al. 2015; Sarstedt and Danks 2022). In our case, we choose a parsimonious specification that accounts for the disclosure of a specific item via indicator variables. Note that our specification tests whether and how the disclosure of *ULCF* and the disclosed value of *ULCF* are associated with future performance outcomes. To rule out potential concerns regarding self-selection, we also perform a robustness check that exclusively considers observations that disclose the mandatorily required information (i.e., see Table A3, Panels A and B, in the Online Appendix C).

17 We also test (untabulated) regressions with two- and four-year-ahead performances with similar findings and implications. The results are available upon request. In our out-of-sample tests in Sects. 4.2 and 4.3, we generally perform forecasts over one, two, three, and four years for earnings and cash flows.

18 For example, we can use data from 2004 to 2012 (with current year performance as the dependent variable and once-lagged explanatory variables) to predict the performance in 2013. By contrast, we only have the sample from 2004 to 2009 to predict the performance in 2010.

19 For example, to predict one-year-ahead cash flows (earnings), the MAFE of the baseline model in Table 5, Panel A multiplied by 100 is 2.93 (2.58), which amounts to 32% (47%) of the average cash flows (earnings) as documented in Table 2, Panel A. By contrast, the corresponding prediction errors of Model NPERF, as reported in Table 5, Panel B, would be 2.95 (2.43) for cash flows (earnings).

20 We find that the error changes of Model NPERF are typically more negative than those of Model NPI, implying lower MAFEs for Model NPERF. In addition, this negative error difference of Model NPERF to Model NPI is statistically significant for predicting two- and three-year-ahead cash flows and for predicting one- to three-year-ahead earnings. We also find that Theil’s U differences are more negative for Model NPERF than for Model NPI. The only relevant exception is the prediction of one-year-ahead cash flows (*CFBT*_*t*+*1*_), where we find increases in the MAFEs and Theil’s U for Model NPERF and reductions for Model NPI. In this case, the differences in the MAFEs of Model NPERF compared to the baseline model are not statistically significant.

21 The adaptive LASSO model selects *D*^*ULCF*^ × *ULCF* in five of eight specifications and the square-root LASSO in only three of eight specifications. Other selected parameters are V*D*^*TLCF*^ × *TLCF* (three of 16 specifications), *D*^*DTL*^ × *TDL* (two of 16 specifications), and *D*^*DTAD*^ × *DTAD* (one of 16 specifications). We do not report which industry and year dummies have been selected.

22 In-sample tests address the question of which estimator fits in the best way a specific data set and therefore explains that data. This also holds for the LASSO method, which minimizes the sum of squared residuals (similar to OLS) but also accounts for a penalty parameter to reduce the number of explanatory variables (Freyberger et al. 2020).

23 Unlike the previous analysis, using BCN as a reference reduces the number of significant changes in MAFEs. Hence, since BCN add controls, it seems less relevant when other variables are included. Nevertheless, we still find that Models ULCF and VD will not enhance performance predictions (and, in some specifications, significantly worsen predictions), while Model NPI and especially Model NPERF significantly reduce MAFEs if compared to the BCN model as the reference.
