# Supplementary material for: Does IFRS information on tax loss carryforwards and negative performance improve predictions of earnings and cash flows?
Source: J Bus Econ. 2023 Apr 29:1–39. Online ahead of print. doi: 10.1007/s11573-023-01147-7 (PMC10148587; doi:10.1007/s11573-023-01147-7)
Supplement: Supplementary file 1 — Supplementary file1 (DOC 514 KB) [file 11573_2023_1147_MOESM1_ESM.doc]

# Online Appendix

# Online Appendix A: Univariate Correlations

Table A1 documents univariate correlations. We consider all variables included in Equation (2) and total assets. We denote statistically significant correlations by ***(1% level), ** (5% level), and * (10% level). As should be expected, we find a strong correlation between our alternative performance indicators, *CFBT* and *EBT*. The same holds for the interaction terms in case of negative performance $NCFBT\times CFBT$and $NEBT\times EBT$. In line with our theoretical expectations, we also find a negative correlation between unrecognized tax loss carryforwards ($D^{ULCF}\times ULCF$) and the performance indicators *CFBT* and *EBT*. Simultaneously, *ULCF* tends to be larger in case of negative performance (*NCFBT* and *NEBT*). The same holds for total loss carryforwards *TLCF* ($D^{TLCF}\times TLCF$) that are also strongly correlated with *ULCF* ($D^{ULCF}\times ULCF$). These associations suggest that firms with higher unrecognized tax loss carryforwards tend to have lower performance and higher total loss carryforwards and vice versa.

[Table A1 about here]

We typically do not find strong associations for the indicator variables regarding the (voluntary) disclosure of information on deferred taxes from tax loss carryforwards. Observations disclosing information on recognized deferred tax assets from loss carryforwards ($D^{DTA LCF}$) have a higher tendency to disclose information on unrecognized tax loss carryforwards ($D^{ULCF}$), and the amount of recognized deferred tax assets from loss carryforwards ($D^{DTA LCF}\times DTA LCF$) is positively correlated with unrecognized loss carryforwards ($D^{ULCF}\times ULCF$). Observations with high deferred tax assets from timing differences ($D^{DTAD}\times DTAD$) have a higher tendency to disclose information on deferred taxes from loss carryforwards ($D^{DTA LCF}$) and to voluntarily disclose a corresponding valuation allowance ($VD^{VAL}$). Deferred tax liabilities ($D^{DTAD}\times DTAD$) are weakly positively correlated to the voluntary disclosure of total loss carryforwards ($VD^{TLCF}$) and the valuation allowance ($VD^{VAL}$).

Table A1. Univariate correlations

| Variables | CFBT | EBT | NCFBT | NEBT | NCFBT × CFBT | NEBT × EBT | D^ULCF^ | D^ULCF^ × ULCF | VD^TLCF^ | VD^TLCF^ × TLCF | VD^VAL^ | VD^VAL^ × VAL | VD^ΔVAL^ | VD^VAL^ × ΔVAL | D^DTA LCF^ | D^DTA LCF^ × DTA LCF | D^DTAD^ × DTAD | D^DTL^ × DTL | Total assets |
| --- | --- | --- | --- | --- | --- | --- | --- | --- | --- | --- | --- | --- | --- | --- | --- | --- | --- | --- | --- |
| CFBT | 1.00 |  |  |  |  |  |  |  |  |  |  |  |  |  |  |  |  |  |  |
| EBT | 0.72** | 1.00 |  |  |  |  |  |  |  |  |  |  |  |  |  |  |  |  |  |
| NCFBT | -0.46** | -0.32** | 1.00 |  |  |  |  |  |  |  |  |  |  |  |  |  |  |  |  |
| NEBT | -0.22** | -0.50** | 0.32** | 1.00 |  |  |  |  |  |  |  |  |  |  |  |  |  |  |  |
| NCFBT × CFBT | 0.40** | 0.39** | -0.57** | -0.26** | 1.00 |  |  |  |  |  |  |  |  |  |  |  |  |  |  |
| NEBT × EBT | 0.23** | 0.59** | -0.31** | -0.49** | 0.62** | 1.00 |  |  |  |  |  |  |  |  |  |  |  |  |  |
| D^ULCF^ | -0.07* | -0.10** | 0.04 | 0.05 | -0.04 | -0.05 | 1.00 |  |  |  |  |  |  |  |  |  |  |  |  |
| D^ULCF^ × ULCF | -0.28** | -0.37** | 0.26** | 0.25** | -0.57** | -0.56** | 0.22** | 1.00 |  |  |  |  |  |  |  |  |  |  |  |
| VD^TLCF^ | 0.17** | 0.06+ | -0.10** | -0.01 | -0.01 | -0.04 | 0.06+ | -0.02 | 1.00 |  |  |  |  |  |  |  |  |  |  |
| VD^TLCF^ × TLCF | -0.19** | -0.35** | 0.22** | 0.29** | -0.50** | -0.54** | -0.06 | 0.57** | 0.32** | 1.00 |  |  |  |  |  |  |  |  |  |
| VD^VAL^ | 0.08* | 0.05 | -0.09* | -0.05 | 0.06 | -0.00 | -0.09* | -0.08* | 0.18** | -0.03 | 1.00 |  |  |  |  |  |  |  |  |
| VD^VAL^ × VAL | -0.05 | -0.07+ | -0.01 | 0.02 | -0.01 | -0.09* | 0.00 | 0.22** | -0.07* | -0.01 | 0.36** | 1.00 |  |  |  |  |  |  |  |
| VD^ΔVAL^ | -0.03 | -0.07+ | 0.06 | 0.03 | 0.02 | -0.02 | 0.09** | -0.03 | 0.04 | -0.03 | 0.11** | 0.03 | 1.00 |  |  |  |  |  |  |
| VD^VAL^ × ΔVAL | -0.03 | -0.07* | 0.04 | 0.10** | 0.00 | -0.10** | 0.00 | 0.08* | -0.01 | 0.04 | 0.00 | 0.02 | 0.31** | 1.00 |  |  |  |  |  |
| D^DTA LCF^ | 0.05 | 0.07* | 0.09** | 0.00 | -0.05 | -0.05 | 0.28** | 0.09** | -0.07+ | -0.01 | 0.10** | 0.01 | 0.09* | 0.03 | 1.00 |  |  |  |  |
| D^DTA LCF^ × DTA LCF | -0.12** | -0.13** | 0.08* | 0.16** | -0.04 | -0.07+ | 0.07* | 0.19** | -0.04 | 0.15** | 0.12** | 0.28** | -0.05 | 0.00 | 0.31** | 1.00 |  |  |  |
| D^DTAD^ × DTAD | 0.11** | 0.05 | -0.03 | -0.00 | 0.05 | -0.01 | 0.12** | 0.01 | 0.04 | -0.03 | 0.30** | 0.16** | 0.06+ | 0.06+ | 0.50** | 0.32** | 1.00 |  |  |
| D^DTL^ × DTL | -0.00 | -0.09** | -0.08* | 0.04 | 0.03 | -0.00 | -0.08* | 0.03 | 0.12** | 0.07+ | 0.11** | -0.02 | -0.08* | -0.04 | -0.16** | 0.12** | 0.36** | 1.00 |  |
| Total assets | -0.21** | -0.14** | 0.07* | 0.02 | 0.03 | 0.05 | 0.11** | -0.08* | -0.09* | -0.08* | -0.03 | -0.06 | 0.15** | -0.04 | 0.05 | -0.12** | -0.12** | -0.16** | 1.00 |
| Univariate correlations between regression variables as well as total assets. We denote statistically significant correlations by ** (1% level), * (5% level) and + (10% level). We provide variable definitions in Table A1. | | | | | | | | | | | | | | | | | | | |

# Online Appendix B: In-sample tests

Our primary test statistic in these tests is an F-test for nested models. We use the same prediction models, as documented in Table 4, Panels A and B (see also Tables 5 and 6). Our analysis aims to determine whether specific models increase the explanatory power of our in-sample regressions. The nested F-test compares the explanatory power of the augmented model, including additional explanatory variables, with the reference model (either Baseline or NPERF). Therefore, it tests which of the models provides more explanatory power. As an alternative statistic, we document in brackets the absolute values of the adjusted R^2^ for each model. For the NPERF Model, we also perform an F-test against the NPI Model [in square brackets]. This is to ensure that the interaction term's inclusion increases explanatory power compared to the reference and NPI models. We report the results in Table A2. Like in Table 5, we test the models in Panel A against the Baseline Model and Panel B against the NPERF Model.

Table A2. In-sample tests

| Model | 1 | 2 | 3 | 4 | | 5 | 6 | 7 | 8 |
| --- | --- | --- | --- | --- | --- | --- | --- | --- | --- |
| Performance measure | Cash flow from operations before taxes | | | | | Earnings before taxes | | | |
| Prediction years | 1 year (CFBT_t+1_) | 2 years (CFBT_t+2_) | 3 years (CFBT_t+3_) | | 4 years (CFBT_t+4_) | 1 year (EBT_t+1_) | 2 years (EBT_t+2_) | 3 years (EBT_t+3_) | 4 years (EBT_t+4_) |
| Panel A: Baseline Model as the reference model | | | | | | | | | |
| Baseline | (0.625) | (0.537) | (0.487) | (0.446) | | (0.593) | (0.381) | (0.302) | (0.231) |
| ULCF | 4.16** | 0.54 | 0.13 | 0.26 | | 0.57 | 0.64 | 1.86 | 1.10 |
|  | (0.628) | (0.537) | (0.486) | (0.445) | | (0.592) | (0.380) | (0.303) | (0.232) |
| VD | 0.60 | 0.86 | 0.50 | 0.79 | | 1.05 | 0.66 | 0.72 | 0.97 |
|  | (0.624) | (0.537) | (0.485) | (0.445) | | (0.593) | (0.379) | (0.300) | (0.231) |
| NPI | 8.22*** | 16.49*** | 5.27** | 3.82* | | 7.50*** | 2.56 | 0.83 | 0.29 |
|  | (0.628) | (0.546) | (0.490) | (0.448) | | (0.596) | (0.382) | (0.302) | (0.231) |
| NPERF | 4.50**  [0.77] | 11.18***  [5.78**] | 4.23**  [3.18*] | 6.19***  [8.52***] | | 12.68***  [17.71***] | 13.44***  [24.25***] | 10.15***  [19.44***] | 6.63***  [12.96***] |
|  | (0.628) | (0.549) | (0.491) | (0.455) | | (0.604) | (0.399) | (0.318) | (0.244) |
| Panel B: NPERF Model as the reference model | | | | | | | | | |
| NPERF | (0.628) | (0.549) | (0.491) | (0.455) | | (0.604) | (0.399) | (0.318) | (0.244) |
| NPULCF | 9.87*** | 4.32** | 1.92 | 1.80 | | 5.37*** | 8.60*** | 11.94*** | 7.97*** |
|  | (0.636) | (0.552) | (0.492) | (0.456) | | (0.608) | (0.410) | (0.337) | (0.259) |
| NPVD | 1.28 | 1.11 | 0.27 | 0.36 | | 2.39** | 2.48** | 2.81** | 3.10*** |
|  | (0.629) | (0.549) | (0.488) | (0.451) | | (0.608) | (0.406) | (0.327) | (0.258) |
| JOINT | 2.20** | 1.33 | 0.86 | 0.94 | | 2.00** | 2.35*** | 2.76*** | 2.52*** |
|  | (0.635) | (0.551) | (0.490) | (0.454) | | (0.610) | (0.411) | (0.336) | (0.264) |
| Observations | 835 | 815 | 792 | 691 | | 835 | 815 | 792 | 691 |
| Panel A shows the adjusted R^2^ (in parentheses) of the Baseline Model and the results of nested F-tests (adjusted R^2^ in parentheses) of the ULCF, VD, NPI, and NPERF Model against the Baseline Model. Panel B shows the adjusted R^2^ (in parentheses) of the NPERF Model, as well as the results of nested F-tests (adjusted R^2^ in parentheses) of the NPULCF, NPVD, and JOINT Models against the NPERF Model. The superscripts ***, **, and * indicate statistically significant nested F-tests compared to the reference model (to the NPI Model in squared brackets) at the 1%, 5%, and 10% levels. Table 4 provides detailed documentation of the prediction models. We provide variable definitions in Table 8 in the Appendix. | | | | | | | | | |

We find no apparent increase in the ULCF and VD prediction models’ explanatory power compared to the Baseline Model. Only in one specification (*CFBT_t+1_*). We find that the explanatory power of ULCF significantly exceeds the power of the Baseline Model. Related to H3, Table A2 suggests that including $NPI\times PERF$ significantly increases explanatory power. The NPERF Model's F-tests against the Baseline are positive and significant. Besides, F-tests of the NPERF Model against the NPI Model are positive and significant in 7 of 8 specifications. Considering the more comprehensive NPULCF, NPVD, and JOINT Models, we find a significant increase in the explanatory power in most specifications compared to the NPERF Model. Thus, enriching the NPERF Model by providing information on unrecognized tax loss carryforwards, deferred taxes, and related items increases explanatory power. This holds especially for earnings as dependent variables. Considering adjusted R^2^, the more comprehensive models with more variables (especially NPULCF and JOINT) provide the highest explanatory power.

# Online Appendix C: Robustness tests

Table A3 provides robustness tests. As mentioned earlier in the paper, there might be a concern that self-selection regarding the (mandatory) disclosure of information on unrecognized tax loss carryforwards (*ULCF*) could affect our results. Therefore, we perform a first robustness check considering the sample disclosing information on *ULCF*. As documented in Table 2, the descriptive statistics of the ULCF Sample do not differ vastly from our full sample. As the indicator variable *D^ULCF^* takes a value of one for all observations in this sample, it will drop out the regressions in this robustness check. Apart from that and the fewer observations, our approach is the same as in the out-of-sample tests in Table 5. The results are documented in Table A3, Panels A, and B, and confirm our previous findings. Again we find that the Models ULCF and VD increase mean forecast errors and Theil’s U if compared to the Baseline Model, while NPI and especially NPERF reduce forecast errors. Furthermore, augmenting NPERF by additional variables leads to significantly larger errors in most specifications (Table A3, Panel B). Thus, the best choice for predictions is NPERF.

Second, one might challenge our straightforward Baseline Model that exclusively considers past performance, year dummies, and industry dummies for predictions (Equation 1). Therefore, we also consider the widely applied model of Barth et al. (2001, in the following BCN) as an alternative reference model for our analysis (see also Bostwick et al. 2016, with further references). This model regresses future cash flow on current cash flow and six accruals: the current year’s change in accounts receivable (*ΔAR*), the change in accounts payable (*ΔAP*), the change in inventories (*ΔINV*), depreciation (*DEPR*), amortization (*AMORT*), and other changes in accruals (*OTHER*), where *OTHER* is the difference in earnings before taxes and operating cash flow adjusted by the five other accrual items *(OTHER = EBT – (CFO + ΔAR – ΔAP + ΔINV + DEPR + AMORT)*. We scale all variables by total assets. While this model has been typically used to predict one-year-ahead cash flows, it can also be used to predict other performance measures. A generalized version of the model is

$\begin{matrix} PERF_{it+x}=\alpha+\beta_{1}\cdot PERF_{it}+\gamma_{1}\cdot\Delta AR_{it}+\gamma_{2}\cdot\Delta AP_{it}+\gamma_{3}\cdot\Delta INV_{it}+\gamma_{4}\cdot DEPR_{it} \\ +\gamma_{5}\cdot AMORT_{it}+\gamma_{6}\cdot OTHER_{it}+\gamma_{7}\cdot INDUSTRY_{i}+\gamma_{8}\cdot YEAR_{t}+u_{it}. \end{matrix}$ (3)

Table A3. Out-of-sample tests: Robustness checks

| Model | 1 | 2 | 3 | 4 | 5 | 6 | 7 | 8 |
| --- | --- | --- | --- | --- | --- | --- | --- | --- |
| Performance measure | Cash flow from operations before taxes | | | | Earnings before taxes | | | |
| Prediction years | 1 year (CFBT_t+1_) | 2 years (CFBT_t+2_) | 3 years (CFBT_t+3_) | 4 years (CFBT_t+4_) | 1 year (EBT_t+1_) | 2 years (EBT_t+2_) | 3 years (EBT_t+3_) | 4 years (EBT_t+4_) |
| Panel A: ULCF Sample and Baseline Model as the reference model | | | | | | | | |
| Baseline absolute | 2.98 | 3.56 | 4.68 | 4.64 | 2.66 | 3.86 | 3.96 | 4.44 |
|  | (13.81) | (16.77) | (34.62) | (38.81) | (13.53) | (33.87) | (35.54) | (45.92) |
| ULCF difference | 0.22** | 0.09* | 0.09 | 0.08* | 0.09** | 0.17** | 0.27** | 0.16 |
|  | (2.41) | (1.09) | (2.38) | (2.15) | (0.67) | (1.31) | (3.91) | (3.35) |
| VD difference | 0.26** | 0.12* | 0.06 | 0.05 | 0.23*** | 0.32** | 0.44*** | 0.32** |
|  | (2.98) | (1.62) | (2.57) | (1.13) | (2.20) | (4.13) | (6.30) | (4.67) |
| NPI difference | -0.02 | -0.01 | -0.07** | -0.02 | -0.11*** | -0.09*** | -0.01 | -0.01 |
|  | (-0.10) | (-0.39) | (-0.23) | (-0.29) | (-0.67) | (-0.47) | (-0.08) | (-0.37) |
| NPERF difference | 0.04 | -0.08 | -0.12** | -0.17 | -0.16^***^/^++^ | -0.25^***/+++^ | -0.25^***/+^ | -0.12 |
|  | (0.94) | (-0.77) | (-0.76) | (-2.34) | (-1.12) | (-2.37) | (-2.82) | (-2.49) |
| Observations | 216 | 204 | 190 | 124 | 216 | 204 | 190 | 124 |
| Panel B: ULCF Sample and NPERF Model as the reference model | | | | | | | | |
| NPERF absolute | 3.06 | 3.48 | 4.56 | 4.47 | 2.50 | 3.61 | 3.72 | 4.33 |
|  | (14.75) | (16.01) | (33.86) | (36.48) | (12.40) | (31.50) | (32.72) | (43.43) |
| NPULCF difference | 0.18*** | 0.19*** | 0.20** | 0.10* | 0.06 | 0.12 | 0.30* | 0.39** |
|  | (1.55) | (0.86) | (1.87) | (0.88) | (0.13) | (0.77) | (4.38) | (5.55) |
| NPVD difference | 0.25*** | 0.22*** | 0.15** | 0.08 | 0.31*** | 0.39*** | 0.58*** | 0.66*** |
|  | (2.26) | (1.75) | (2.32) | (1.23) | (2.35) | (5.95) | (9.12) | (9.06) |
| JOINT difference | 0.32*** | 0.30*** | 0.24** | 0.16* | 0.38*** | 0.35** | 0.51** | 0.73*** |
|  | (3.01) | (1.96) | (2.68) | (1.59) | (2.49) | (5.76) | (10.07) | (10.17) |
| Observations | 216 | 204 | 190 | 124 | 216 | 204 | 190 | 124 |
| Panel C: BCN Model as the reference model | | | | | | | | |
| BCN absolute | 3.28 | 3.88 | 5.37 | 5.25 | 2.87 | 3.85 | 3.89 | 3.84 |
|  | (14.65) | (19.27) | (46.24) | (53.88) | (21.41) | (36.27) | (36.48) | (41.94) |
| ULCF difference | 0.12* | 0.00 | 0.03 | 0.04 | 0.04* | 0.05 | 0.06 | 0.07 |
|  | (1.74) | (0.31) | (0.95) | (1.03) | (0.57) | (1.03) | (2.26) | (2.50) |
| VD difference | 0.04 | 0.04 | -0.04 | -0.09 | 0.20*** | 0.06 | 0.10 | 0.24** |
|  | (0.68) | (0.53) | (-0.36) | (-1.62) | (1.35) | (1.53) | (1.76) | (3.23) |
| NPI difference | -0.11 | -0.15 | -0.12 | -0.11 | -0.02** | 0.00 | -0.02 | 0.00 |
|  | (-0.23) | (-1.95) | (-2.95) | (-2.42) | (-.06) | (-0.02) | (-0.03) | (-0.04) |
| NPERF difference | 0.02 | -0.06 | -0.11** | -0.18* | -0.12^***/++^ | -0.17^***/+++^ | -0.12^**/+^ | -0.02 |
|  | (2.23) | (-0.67) | (-0.88) | (-3.71) | (-1.13) | (-1.19) | (-1.15) | (-1.33) |
| Observations | 220 | 215 | 209 | 140 | 220 | 215 | 209 | 140 |
| Panel D: BCN NPERF Model as reference model | | | | | | | | |
| BCN NPERF absolute | 3.30 | 3.83 | 5.26 | 5.07 | 2.75 | 3.67 | 3.77 | 3.86 |
|  | (16.88) | (18.60) | (45.36) | (50.17) | (20.29) | (35.09) | (35.33) | (40.61) |
| NPULCF difference | 0.07 | 0.07 | 0.03 | -0.02 | 0.07 | 0.33** | 0.19 | 0.27 |
|  | (0.12) | (0.14) | (-0.26) | (-1.06) | (0.61) | (3.39) | (5.30) | (4.65) |
| NPVD difference | 0.05 | 0.08 | -0.06 | -0.10* | 0.21*** | 0.26** | 0.20* | 0.26* |
|  | (0.39) | (0.81) | (-0.59) | (-1.75) | (1.55) | (2.57) | (2.81) | (3.58) |
| JOINT difference | 0.10 | 0.24* | -0.08 | -0.28** | 0.42*** | 0.50* | 0.29 | 0.71** |
|  | (0.70) | (1.83) | (-1.61) | (-5.04) | (4.72) | (10.10) | (12.23) | (12.89) |
| Observations | 220 | 215 | 209 | 140 | 220 | 215 | 209 | 140 |
| Panel A and B report results for the ULCF Sample, while Panel C and D report results for the full sample and the BCN Model. Panel A (C) shows the absolute mean forecasting errors (MAFEs) of the Baseline Model (BCN Model) and corresponding Theil´s U statistics in parentheses, as well as the differences of MAFEs (differences of Theil´s U statistics in parentheses) of the ULCF, VD, NPI, and NPERF Models compared to the Baseline Model BCN Model). Panel B (D) shows the absolute MAFEs (Theil’s U statistics in parentheses) of the NPERF Model (BCN NPERF Model) and the differences of MAFEs (differences of Theil´s U statistics in parentheses) of the NPULCF, NPVD, and JOINT Models compared to the NPERF Model (BCN NPERF Model). The superscripts ***, **, and * (^+++^, ^++^, and ^+^) indicate statistically significa88t differences in MAFEs compared to the reference model (to the NPI Model) at the 1%, 5%, and 10% levels. Table 4 provides detailed documentation of the prediction models. We provide variable definitions in Table 8 in the Appendix. | | | | | | | | |

Due to missing information, using the BCN Model as an alternative reference point reduces observations. The results are documented in Table A3, Panels C, and D and widely confirm our previous findings. Unlike previous analyses, using BCN as a reference point reduces the number of significant changes in MAFEs. Hence, as BCN adds controls, it seems less relevant when other variables are included. Nevertheless, we still find that ULCF and VD will not enhance performance predictions (and, in several specifications, significantly worsen predictions), while NPI and especially NPERF significantly reduce MAFEs if compared to the BCN Model as a point of reference. In the case of NPERF, we observe significant reductions of errors in 5 of 8 specifications. An interesting further observation is that absolute average errors using the BCN Model seem higher than in the simpler Baseline Model. For example, the absolute BCN error (multiplied as usual by 100) for one-year-ahead cash flow in Panel C is 3.28. The absolute Baseline error (multiplied by 100) for one-year-ahead cash flow in Panel A is 2.98. This finding fits well with Lev et al. (2010) and supports the view that variables based on estimates and subject to discretion may increase forecast errors. Nevertheless, as the samples of Panel A and Panel C differ, the outcome should be interpreted cautiously.

In Panel D, we compare the BCN Model enriched by *NPI* and *NPI × NPERF* (BCN NPERF) with augmented models using additional information from Equation (2). While we find significantly larger forecasting errors for earnings, some results suggest a significant reduction of forecasting errors for four-year-ahead cash flows. While the results are mixed and are not very robust, we may still conclude that we do not find evidence that NPULCF, NPVD, or JOINT significantly reduce prediction errors.

# Online Appendix D: Model extensions

We further extend our analyses by several additional variables. We intend to test whether these variables increase predictive validity (out-of-sample tests). Joos and Plesko (2005) and Li (2011) develop models to predict loss reversals. These models predict the probability that firm–years with (persistent) negative performance in previous years retain positive earnings or cash flows in the current period. As a measure of persistent negative performance, both models rely on count variables for a sequence of persistent negative performance and find a positive impact on the probability of future negative performance. Two other important variables for identifying persistent negative performance in these models are an indicator variable for the firm–years with a first negative performance year and a variable for firms paying out a dividend in the current year. The authors find that firm–years with a sequence of adverse performance outcomes have a lower probability of loss reversal in past years. In comparison, first-year negative performance and dividend payment are indicators of a higher likelihood of loss reversal. Forecasting models also consider indicator variables for dividend payments for expected earnings in the tradition of Fama and French (2000; see, e.g., Fama and French 2006; Hou et al. 2012).

To account for sequences of years with negative performance, we enrich the model by $D^{NSEQ}$ and $D^{NSEQ}\times NSEQ$. $D^{NSEQ}$ accounts for whether information on past negative performance is available. We only consider information from IFRS accounts. We regard the information on loss persistence as missing and set the value of $D^{NSEQ}$ to zero in case of a change in accounting methods. The term $D^{NSEQ}\times NSEQ$ is the interaction of $D^{NSEQ}$ and a variable *NSEQ* with a value of one for negative firm performance in the last year (earnings or cash flows, depending on the performance measure *PERF*), a value of two for negative performance in the two last years, and a value of three for negative performance in the three last years. In all other cases, *NSEQ* takes a value of zero. In our standard specification, a positive value of *NSEQ* does not require a loss in the current period. In unreported tests (available upon request), we test an alternative specification of *NSEQ (NSEQA)*, where only firm–years with negative performance in the current period and a sequence of negative performance in previous periods are considered (past and present negative performance). *NSEQA* has a value of one to three if performance is negative in the current year and one to three preceding years. This alternative specification typically leads to higher forecasting errors than our standard specification of *NSEQ*. Thus, we abstain from reporting results. As for indicators for transitory negative performance, we also test an indicator variable for the firm–years with a first-time negative performance (*FNPI*) (that means no negative performance in the preceding period) and a variable *DIV* defined as the ratio of the cash dividend (Worldscope item 04551) and total assets.[[1]](#footnote-2) Like our standard procedure, we enrich the Model NPERF by indicator variables with zero value if the corresponding information is unavailable (*D^FNPI^*, *D^DIV^*) and interaction terms ($D^{FNPI}\times FNPI,$ $D^{DIV}\times DIV$).

Following a suggestion of one of our reviewers, we also test a parameter on relative negative performance (i.e., a reduction in the performance parameter (earnings or cash flows) compared to the preceding period) as an additional measure for transitory losses. The indicator variable *RNPI* takes a value of one if the current performance is smaller than the performance in the previous year. Again, we enrich the Model NPERF by an indicator variable with zero value if the corresponding information is unavailable (*D^RNPI^*) and a related interaction term ($D^{RNPI}\times RNPI$).

Another relevant issue is the information content of R&D for profit and, especially, loss firms. Theory and empirical evidence suggest that R&D expenditures are related to (uncertain) future earnings and value relevance (Kothari et al. 2002). Since R&D is a business investment (Ciftci and Darrough 2015; Darrough and Ye 2007), one might expect higher future earnings and cash flows for high R&D firm–years. Besides, high R&D levels could be an indicator of future loss reversal. Adding information on R&D to the prediction model could enhance predictive validity. We enrich the model with an indicator variable for firm information on R&D expenditures ($D^{RD}$), an interaction term of R&D expenditures scaled by total assets ($D^{RD}\times RD$), and an additional interaction term with our indicator variable for loss firms to control for different R&D effects for the firm–years with negative performance and firm–years with positive performance ($NPI \times D^{RD} \times RD$).

Furthermore, we test whether considering the market-to-book ratio (*MTB*) or firm size (*SIZE*) improves predictive validity. We measure *MTB* as the ratio of the market value of the firm at year-end (Worldscope item 07210) to the book value of equity (Worldscope item 07220) and consider an indicator variable for a firm’s information on *MTB* (*D^MTB^*) and an interaction term ($D^{MTB}\times MTB$). We define *SIZE* as the logarithm of total assets. Since all our sample observations provide this information, we do not consider a disclosure dummy or an interaction term in this case.

Untabulated regression results confirm our expectations and the findings of the literature (Darrough and Ye 2007; Joos and Plesko 2005; Kothari et al. 2002; Li 2011) that *FNPI*, *DIV*, *RD*, and *MTB* are positively associated with future firm performance, while *NSEQ* is negatively related to future performance. Since our interest is in these variables' predictive validity, we concentrate on our out-of-sample tests compared to our best-practice model, NPERF. We also calculated (unreported) tests against the Baseline Model with consistent results. The specifications of these tests are the same as in the previous subsamples. We test the following models in addition to our NPERF Model: The NPNSEQ, NPFNPI, NPRNPI, NPDIV, NPRD, and NPMTB, NPSIZE Models. We document the models' detailed definitions and the variables' definitions in Table A4.

**Table A4.** Additional variables and models for out-of-sample tests

| Panel A: Variables |  |
| --- | --- |
| Variable | Definition |
| D^NSEQ^ | Indicator variable with a value of one if the information on NSEQ is available (information on PERF_t-1_, PERF_t-2_, and PERF_t-3_ available). |
| D^NSEQ^ × NSEQ | Interaction term of D^NSEQ^ and NSEQ, where NSEQ documents the sequence of perpetual years with negative firm performance (either earnings or cash flows as a performance measure) in the last three years. It can have values of one (negative performance NPERF in year t - 1), two (NPERF in years t - 1 and t - 2), and three (NPERF in years t - 1, t - 2, and t - 3). |
| D^FNPI^ | Indicator variable with a value of one if the information on FNPI is available. |
| D^FNPI^ × FNPI | Interaction term of D^FNPI^ and FNPI, where the indicator variable FNPI has a value of one if the firm has a first-time negative performance (i.e., negative earnings or cash flows) in the current period, with non-negative earnings or cash flows in the previous period. |
| D^RNPI^ | Indicator variable with a value of one if the information on RNPI is available. |
| D^RNPI^ × RNPI | Interaction term of D^FNPI^ and RNPI, where the indicator variable RNPI has a value of one if the firm has a relative negative performance (i.e., lower earnings or cash flows) compared to the performance in the last period |
| D^DIV^ | Indicator variable with a value of one if the information on cash dividends is available. |
| D^DIV^ × DIV | Interaction term of D^DIV^ and cash dividends scaled by total assets. |
| D^RD^ | Indicator variable with a value of one if the information on research & development expenses is available. |
| D^RD^ × RD | Interaction term of D^RD^ and research & development expenses scaled by total assets. |
| D^RD^ × RD × NPI | Interaction term of D^RD^, research & development expenses scaled by total assets, and NPI. |
| D^MTB^ | Indicator variable with a value of one if the information on MTB is available. |
| D^MTB^ × MTB | Interaction term of D^MTB^ and the ratio of the market capitalization at the end of the year to the book value of equity. |
| SIZE | Logarithm of total assets. |
| Panel B: Models | |
| Model | Definition |
| NPNSEQ | NPERF Model enriched by variables on a sequence of negative performance: D^NSEQ^ and D^NSEQ^ × NSEQ. |
| NPFNPI | NPERF Model enriched by variables on first-time negative performance: D^FNPI^ and D^FNPI^ × FNPI. |
| NPRNPI | NPERF Model enriched by variables on relative negative performance (compared to the previous year): D^RNPI^ and D^RNPI^ × RNPI. |
| NPDIV | NPERF Model enriched by variables on dividend payments: D^DIV^ and D^DIV^ × DIV. |
| NPRD | NPERF Model enriched by variables on research and development expenses: D^RD^, D^RD^ × RD, and D^RD^ × RD × NPI. |
| NPMTB | NPERF Model enriched by variables on the market-to-book ratio: D^MTB^ and D^MTB^ × MTB. |
| NPSIZE | NPERF Model enriched by firm size: SIZE. |
| Documentation of models for additional out-of-sample tests. Detailed variable definitions are also provided in Table 8 in the Appendix. | |

Table A5 documents the estimates of our out-of-sample tests. Similar to Tables 6 and 7, additional variables do not necessarily enhance predictive validity. For the NPRD model, we even find significantly higher forecasting errors in most specifications. Thus, adding information on R&D expenditures reduces the predictive validity of our models. Regarding NPFNPI and NPDIV, we obtain inconclusive results. Thus, including variables on first-time negative performance or dividends could either reduce or increase forecasting errors. Including information on the market-to-book ratio (NPMTB) typically has a positive but not statistically significant effect on predictive validity. Only in one of the 8 model specifications we find a reduction of the forecasting error being statistically significant at the 10% level. The only models that typically enhance predictive validity if compared to the NPERF Model are NPSIZE and (with less clear evidence) NPNSEQ and NPRNPI. For NPNSEQ and NPRNPI, we find one specification (in both cases with four-years-ahead earnings as the dependent variable) with a significant increase in MAFE. For NPSIZE, we find a significant reduction in forecasting errors in half of the specifications and never a significant increase.

Concluding, adding information on firm size (measured by the logarithm of total assets), as well as adding information on the sequence of past years with negative performance (*NSEQ*) or relative changes in performance (*RNPI*) rather enhances predictive validity. In unreported tests, we also analyze variables for extraordinary items (Li 2011) and alternative specifications for dividend-paying firms and firm–years with a sequence of past losses or negative cash flows with virtually unchanged results.

Table A5. Additional analyses, out-of-sample tests

| Model | 1 | 2 | 3 | 4 | 5 | 6 | 7 | 8 |
| --- | --- | --- | --- | --- | --- | --- | --- | --- |
| Performance measure | Operating cash flow | | | | Earnings before taxes | | | |
| Prediction years | 1 year (CFBT_t+1_) | 2 years (CFBT_t+2_) | 3 years (CFBT_t+3_) | 4 years (CFBT_t+4_) | 1 year (EBT_t+1_) | 2 years (EBT_t+2_) | 3 years (EBT_t+3_) | 4 years (EBT_t+4_) |
| NPERF absolute | 2.95 | 3.42 | 4.48*** | 4.58** | 2.43*** | 3.64*** | 3.87*** | 4.06 |
|  | (17.35) | (19.44) | (41.23) | (49.79) | (21.51) | (38.42) | (41.62) | (50.67) |
| NPNSEQ difference | 0.00 | 0.04 | 0.04 | -0.10** | -0.01 | -0.23*** | -0.25*** | 0.10* |
|  | (-0.04) | (0.05) | (-0.09) | (-1.63) | (-0.13) | (-1.72) | (-1.28) | (1.11) |
| NPFNPI difference | 0.01 | 0.43*** | 0.29 | -0.21 | 0.13*** | -0.46*** | -0.38*** | 0.89*** |
|  | (0.33) | (3.02) | (0.20) | (-4.99) | (1.85) | (-2.75) | (-1.00) | (9.64) |
| NPRNPI difference | 0.04 | -0.02 | -0.09** | -0.09 | -0.00 | -0.01*** | -0.14*** | 0.03** |
|  | (-0.65) | (-0.10) | (-1.84) | (-2.35) | (-0.09) | (-0.09) | (-0.86) | (0.34) |
| NPDIV difference | -0.02 | 0.00 | 0.02 | 0.08** | 0.01* | -0.00 | 0.00 | 0.00 |
|  | (-0.16) | (-0.03) | (0.28) | (1.13) | (0.01) | (-0.01) | (-0.01) | (0.30) |
| NPRD difference | 0.02 | 0.03 | 0.05 | 0.08 | 0.03 | 0.10** | 0.11** | -0.02 |
|  | (0.14) | (0.51) | (0.64) | (1.27) | (0.03) | (0.65) | (1.91) | (1.74) |
| NPMTB difference | 0.09 | 0.19 | 0.13 | -0.02 | 0.07 | 0.09 | -0.20* | 0.02 |
|  | (2.86) | (9.92) | (2.87) | (2.00) | (8.89) | (19.94) | (0.18) | (-0.20) |
| NPSIZE difference | -0.01 | -0.03** | -0.08** | -0.05 | -0.01 | -0.02*** | -0.02*** | 0.00 |
|  | (-0.04) | (-0.07) | (-0.74) | (-0.86) | (-0.03) | (-0.13) | (-0.07) | (-0.02) |
| Observations | 285 | 271 | 255 | 167 | 285 | 271 | 255 | 167 |
| This table shows the absolute MAFEs (Theil’s U statistics in parentheses) of the NPERF Model and the differences of MAFEs (differences of Theil´s U statistics in parentheses) of the NPNSEQ, NPFNPI, NPRNPI, NPDIV, NPRD, NPMTB, and NPSIZE Models compared to the NPERF Model. The superscripts ***, **, and * indicate statistically significant differences in MAFEs compared to the NPERF Model at the 1%, 5%, and 10% levels. Panel B of Tables 5 and A4 provides detailed documentation of the prediction models. We provide variable definitions in Table 8 and in Table A3, Panel A. | | | | | | | | |

**Online Appendix E: Industry interaction terms and trends**

Economic trends and developments can differ between industries. Therefore, an interesting question is whether considering industry-specific relationships among variables might help improve predictions. As documented by previous analyses, the best performing model is NPERF. Thus, we use this model as a starting point. We interact all main explanatory variables of this model (*PERF*_it_, NPI_it_, and *PERF*_it_×NPI_it_) with either a) dummy variables for industries or b) linear industry trends. As trend indicators, we use the survey years (i.e., 2,004 for 2004).[[2]](#footnote-3) Thus, in the industry-adjusted model NPERF INDUSTRY, we have an industry-specific version of *PERF*_it_, NPI_it_, and *PERF*_it_×NPI_it_, for all industries, and in the NPERF INDTREND model, interactions terms of all variables with industry-specific trends. Table A6 provides an overview of our industry-specific models.

Table A6. Models with industry interaction terms

| Model | Definition |
| --- | --- |
| NPERF INDUSTRY | NPERF Model enriched by interaction terms with industry dummy variables. |
| NPERF INDTREND | NPERF Model enriched by interaction terms with industry trends. |
| NPULCF INDUSTRY | NPULCF Model enriched by interaction terms with industry dummy variables. |
| NPULCF INDTREND | NPULCF Model enriched by interaction terms with industry trends. |
| LASSO INDUSTRY | Starting with the JOINT model enriched by interaction terms with industry dummies, we apply the adaptive LASSO method to obtain an optimal prediction model. |
| LASSO INDTREND | Starting with the JOINT model enriched by interaction terms with industry trends, we apply the adaptive LASSO method to obtain an optimal prediction model. |
| Documentation of the prediction models. We provide detailed variable definitions in Table 8 in the Appendix. | |

As our focus is also on the analysis of unrecognized loss carryforwards (ULCF), we further consider industry-adjusted versions of the NPULCF Model that enriches NPERF by the variables $D_{it}^{ULCF}$ and $_{it}^{ULCF}\times ULCF_{it}$ (NPULCF INDUSTRY and NPULCF INDTREND). In principle, we could also interact all Equation (2) variables with industry indicators or trends, resulting in an industry-specific version of the model JOINT. However, a problem with such a specification is that it results in many explanatory variables. As already documented by the model JOINT, a very high number or predictors may increase prediction errors if associations between variables are unstable (model overfitting). Therefore, we use an industry-adjusted version of JOINT as a starting point and then apply the adaptive LASSO method to reduce the number of explanatory variables. In doing so, we either use a) industry dummy variables or b) industry trends to calculate interactions. We test all these models against NPERF as the baseline model and present out-of-sample results in Table A7.

Results document that interactions with industry trends are not useful to reduce forecasting errors. We find almost no specification that reduces Theil’s U or MAFEs in such models (only for the LASSO INDTREND model, which probably does not select trend variables). In addition, we also find high increases in Theil’s U statistics in some models that point to considerable noise in measurement errors. Indeed, Theil’s U can increase by more than 10,000% in earnings forecasts (see NPERF INDTREND and NPULCF INDTREND). The main problem should be that trends often change over time. However, this is hard to foresee in a prediction model. While we assume a linear trend, the underlying functional form of trends is uncertain and might also change over time.

Regarding interactions with industry dummies, the evidence is less clear. While LASSO INDUSTRY and NPULCF INDUSTRY, in most specifications, suggest a reduction of predictive validity, we do not find any statistically significant increase of MAFEs for the NPERF INDUSTRY model. For four-years-ahead earnings, we also state a significant reduction of MAFEs of NPERF INDUSTRY compared to NPERF. Theil’s U statistics of this model suggest that industry interactions may enhance predictive validity for earnings (lower Theil’s U in models 5 to 8) but reduce predictive validity for cash flows (higher Theil’s U in models 1 to 4). Overall, our evidence suggests that industry interaction terms can be useful for the prediction of earnings but not for cash flows. Industry trends worsen predictions.

Table A7. Industry interaction terms

| Model | 1 | 2 | 3 | 4 | 5 | 6 | 7 | 8 |
| --- | --- | --- | --- | --- | --- | --- | --- | --- |
| Performance measure | Cash flow from operations | | | | Earnings before taxes | | | |
| Prediction years | 1 year (CFBT_t+1_) | 2 years (CFBT_t+2_) | 3 years (CFBT_t+3_) | 4 years (CFBT_t+4_) | 1 year (EBT_t+1_) | 2 years (EBT_t+2_) | 3 years (EBT_t+3_) | 4 years (EBT_t+4_) |
| NPERF absolute | 2.95 | 3.42 | 4.48+ | 4.58 | 2.43 | 3.64 | 3.87 | 4.06 |
|  | (17.35) | (19.44) | (41.23) | (49.79) | (21.51) | (38.42) | (41.62) | (50.67) |
| NPERF INDUSTRY | 0.09 | -0.01 | 0.07 | 0.01 | 0.00 | -0.00 | 0.03 | -0.20* |
| difference | (4.00) | (1.05) | (2.81) | (1.50) | (-0.12) | (-0.24) | (-0.76) | (-4.63) |
| NPERF INDTREND | 0.79*** | 0.18** | -0.17 | 0.37*** | 1.03 | 1.93 | 3.07 | 2.15 |
| difference | (6.50) | (2.45) | (-1.71) | (6.30) | (22,027.84) | (46,545.98) | (133,267.11) | (66,514.65) |
| NPULCF INDUSTRY | 0.26** | 0.18* | 0.20** | 0.14 | 0.13* | 0.29** | 0.27 | -0.15 |
| Difference | (4.68) | (2.59) | (4.43) | (3.02) | (0.94) | (4.36) | (5.76) | (0.39) |
| NPULCF INDTREND | 0.72*** | 0.31*** | -0.12 | 0.41** | 1.29 | 1.91 | 3.18 | 2.35 |
| difference | (6.36) | (3.66) | (-0.45) | (6.82) | (42,529.84) | (41,454.72) | (138,354.63) | (78,313.34) |
| LASSO INDUSTRY | 0.28*** | 0.15* | 0.19** | 0.39*** | 0.09 | -0.34*** | -0.22 | 0.09 |
| difference | (1.55) | (1.55) | (2.41) | (6.82) | (0.24) | (-1.94) | (3.80) | (2.27) |
| LASSO INDTREND | 0.08 | 0.04 | 0.20*** | 0.37*** | 0.13* | -0.24* | -0.23 | -0.07 |
| difference | (1.00) | (0.94) | (2.41) | (6.26) | (1.78) | (-0.47) | (2.49) | (-0.03) |
| Observations | 285 | 271 | 255 | 167 | 285 | 271 | 255 | 167 |
| The table shows the absolute MAFEs and Theil’s U statistics in parentheses (both multiplied by 100) of the NPERF Model and the differences of MAFEs as well as differences of Theil´s U statistics in parentheses (both multiplied by 100) of the NPERF INDUSTRY, NPERF INDTREND, NPULCF INDUSTRY, NPULCE INDTREND, LASSO INDUSTRY, and LASSO INDTREND Models compared to the NPERF Model. The superscripts ***, **, and * (^+++^, ^++^, and ^+^) indicate statistically significant differences in MAFEs compared to the reference model (to the NPI Model) at the 1%, 5%, and 10% levels. Table A6 provides detailed documentation of the prediction models. We provide variable definitions in Table 8 in the Appendix. | | | | | | | | |

#

**Online Appendix F: Coefficients of dummy variables and interaction terms**

To account for the missing information in our data (that also arises in real prediction cases, as information might not be disclosed), we divide our measure for ULCF (similar to other accounting items) into two variables. The following Online Appendix discusses how the corresponding coefficient might be interpreted. *D^ULCF^* measures the difference between disclosing and non-disclosing firms CONDITIONAL on disclosing firms having ULFC = 0. This is because we subdivide the effect of ULCF reporting into two effects considered by two distinct variables:

- The first variable (*D^ULCF^)* captures the effect of reporting versus not reporting ULCF (regardless of the reported value). We find that the association of this disclosure effect with future firm performance is not statistically significant (see Table 3).
- The second effect (denoted by *D^ULCF^ x ULCF*) captures the impact of the reported value of ULCF.

As the reported value must of ULCF be positive (also suggesting a positive mean value of ULCF), the aggregate effect of a firm reporting ULCF compared to a firm not reporting ULCF results from the sum of both a) the disclosure effect (denoted by *D^ULCF^*) and the significantly negative impact of the reported value (indicated by *D^ULCF^ x ULCF*).

*D^ULCF^* can be regarded as an endogenous variable. However, this is not a problem for our analysis. First, as already mentioned, this endogeneity concern holds for most financial account variables.[[3]](#footnote-4) Second and more relevant, as predictions are conceptually different from explanatory models (Hagerty and Srinivasan 1991; Shumeli 2010; Sarstedt and Danks 2022), they do not require exogenous parameters. Indeed, a prediction does not intend to identify a causal relationship or even the coefficient of a specific parameter. Instead, a prediction intends to identify modeling choices or parameter choices that minimize prediction errors. This is also the intention of our model, and the analysis of the MAFEs is a sufficient statistic to analyze if the models meet our targets or not. From this perspective, even the question of how specific coefficients should be interpreted is not the central issue of the paper.

In the following, we provide evidence that the separation of the ULCF effect in the two variables *D^ULCF^* and *D^ULCF^ x ULCF* does not induce a relevant bias into our model. Indeed, we show that the primary results do not change for difference specifications of both variables. First of all, we present our baseline regression in Table A8 for our standard specification but also for the BCN model from Online Appendix C.

**Table A8:** Baseline model

| Model | 1 | 2 | 3 | 4 | 5 | 6 | 7 | 8 |
| --- | --- | --- | --- | --- | --- | --- | --- | --- |
| Performance measure | Cash flow | | | | Earnings before taxes | | | |
| Reference model | Baseline | | BCN | | Baseline | | BCN | |
| Dependent variable | CFBT_t+1_ | CFBT_t+3_ | CFBT_t+1_ | CFBT_t+3_ | EBT_t+1_ | EBT_t+3_ | EBT_t+1_ | EBT_t+3_ |
| PERF | 0.732*** | 0.570*** | 0.844*** | 0.670*** | 0.832*** | 0.577*** | 0.880*** | 0.718*** |
|  | (0.057) | (0.071) | (0.054) | (0.056) | (0.066) | (0.144) | (0.061) | (0.078) |
| NPI | 0.013 | 0.009 | 0.011 | 0.000 | 0.010 | -0.008 | -0.001 | -0.010 |
|  | (0.010) | (0.020) | (0.021) | (0.036) | (0.010) | (0.017) | (0.014) | (0.021) |
| NPI × PERF | -0.442*** | -0.448* | -0.619*** | -0.797** | -0.378*** | -0.659*** | -0.427*** | -0.729*** |
|  | (0.164) | (0.252) | (0.199) | (0.360) | (0.124) | (0.182) | (0.095) | (0.190) |
| D^ULCF^ | -0.006 | 0.000 | 0.001 | 0.004 | 0.004 | 0.004 | 0.001 | -0.001 |
|  | (0.004) | (0.007) | (0.005) | (0.007) | (0.005) | (0.009) | (0.005) | (0.008) |
| D^ULCF^ × ULCF | -0.062** | -0.059** | -0.073** | -0.095*** | -0.042** | -0.095** | -0.050*** | -0.126*** |
|  | (0.028) | (0.027) | (0.030) | (0.033) | (0.021) | (0.045) | (0.017) | (0.036) |
| DTA and VD variables | YES | YES | YES | YES | YES | YES | YES | YES |
| BCN controls | NO | NO | YES | YES | NO | NO | YES | YES |
| Industry effects | YES | YES | YES | YES | YES | YES | YES | YES |
| Year effects | YES | YES | YES | YES | YES | YES | YES | YES |
| Observations | 835 | 792 | 646 | 624 | 835 | 792 | 646 | 624 |
| R^2^ | 0.647 | 0.508 | 0.653 | 0.512 | 0.623 | 0.359 | 0.647 | 0.452 |
| Adjusted R^2^ | 0.635 | 0.490 | 0.635 | 0.486 | 0.610 | 0.336 | 0.629 | 0.422 |
| The dependent variable is operating cash flow before taxes CFBT at time *t* + 1, CFBT at time *t* + 3, earnings before taxes EBT at time *t* + 1, or EBT at time *t* + 3 (in each case scaled by total assets). Estimates are calculated by OLS. Heteroscedasticity-robust standard errors are clustered at the firm level and documented in parentheses. The superscripts ***, **, and * indicate statistical significance at the 1%, 5%, and 10% levels, respectively. The term PERF is the current value of the dependent variable. Table 8 in the Appendix provides detailed variable definitions. | | | | | | | | |

**Table A9:** Exclusively ULCF dummy variable

| Model | 1 | 2 | 3 | 4 | 5 | 6 | 7 | 8 |
| --- | --- | --- | --- | --- | --- | --- | --- | --- |
| Performance measure | Cash flow | | | | Earnings before taxes | | | |
| Reference model | Baseline | | BCN | | Baseline | | BCN | |
| Dependent variable | CFBT_t+1_ | CFBT_t+3_ | CFBT_t+1_ | CFBT_t+3_ | EBT_t+1_ | EBT_t+3_ | EBT_t+1_ | EBT_t+3_ |
| PERF | 0.733*** | 0.571*** | 0.850*** | 0.680*** | 0.830*** | 0.575*** | 0.881*** | 0.723*** |
|  | (0.057) | (0.070) | (0.053) | (0.054) | (0.066) | (0.144) | (0.061) | (0.078) |
| NPI | 0.016* | 0.010 | 0.015 | 0.000 | 0.011 | -0.006 | -0.000 | -0.007 |
|  | (0.009) | (0.020) | (0.021) | (0.039) | (0.010) | (0.017) | (0.015) | (0.023) |
| NPI × PERF | -0.254** | -0.299 | -0.308*** | -0.480 | -0.331*** | -0.543*** | -0.339*** | -0.482** |
|  | (0.117) | (0.242) | (0.116) | (0.405) | (0.117) | (0.169) | (0.123) | (0.230) |
| D^ULCF^ | -0.011** | -0.004 | -0.005 | -0.002 | 0.001 | -0.003 | -0.003 | -0.009 |
|  | (0.004) | (0.007) | (0.005) | (0.007) | (0.006) | (0.009) | (0.005) | (0.008) |
| DTA and VD variables | YES | YES | YES | YES | YES | YES | YES | YES |
| BCN controls | NO | NO | YES | YES | NO | NO | YES | YES |
| Industry effects | YES | YES | YES | YES | YES | YES | YES | YES |
| Year effects | YES | YES | YES | YES | YES | YES | YES | YES |
| Observations | 835 | 792 | 646 | 624 | 835 | 792 | 646 | 624 |
| R^2^ | 0.643 | 0.504 | 0.648 | 0.505 | 0.621 | 0.350 | 0.645 | 0.442 |
| Adjusted R^2^ | 0.631 | 0.487 | 0.630 | 0.479 | 0.608 | 0.327 | 0.627 | 0.412 |
| The dependent variable is operating cash flow before taxes CFBT at time *t* + 1, CFBT at time *t* + 3, earnings before taxes EBT at time *t* + 1, or EBT at time *t* + 3 (in each case scaled by total assets). Estimates are calculated by OLS. Heteroscedasticity-robust standard errors are clustered at the firm level and documented in parentheses. The superscripts ***, **, and * indicate statistical significance at the 1%, 5%, and 10% levels, respectively. The term PERF is the current value of the dependent variable. Table 8 in the Appendix provides detailed variable definitions. | | | | | | | | |

In Table A9 we show results for an alternative specification with only the dummy variable *D^ULCF^* but excluding the interaction term *D^ULCF^ x ULCF*. Our results show that we typically do also not find a significant difference between firms reporting and not reporting ULCF (captured by *D^ULCF^*) if the interaction term *D^ULCF^ x ULCF* is not included. The only exception is the Baseline Model with one-year-ahead cashflows, where we find a significantly lower performance of firms not reporting ULCF if we do not include the interaction term *D^ULCF^ x ULCF*. Thus, we do not find much evidence that reporting (or not reporting) ULCF is indicative of future firm performance.

Table A9 does not mean that the coefficient of the interaction term *D^ULCF^ x ULCF* remains unaffected by the exact specification of the dummy variable *D^ULCF^* that captures in our baseline specification the difference between firms not disclosing ULCF compared to firms disclosing ULCF of zero. Therefore, we perform an additional test that uses a means-adjusted version of *D^ULCF^ x ULCF*. In this specification, we define *ULCF Mean* as the difference between its baseline value (unrecognized tax loss carryforwards divided by total assets) and the sample mean for observations reporting ULCF. Hence, the average value of *ULCF Mean* is zero, and firms with abnormally high (abnormally low) ULCF values have a positive (negative) value of *ULCF Mean*. In this specification, *D^ULCF^* captures the difference in the future firm performance of firms not reporting ULCF and firms reporting the average value of ULCF for firms disclosing ULCF. We report corresponding results in Table A10.

**Table A10:** Mean-adjusted ULCF interaction term

| Model | 1 | 2 | 3 | 4 | 5 | 6 | 7 | 8 |
| --- | --- | --- | --- | --- | --- | --- | --- | --- |
| Performance measure | Cash flow | | | | Earnings before taxes | | | |
| Reference model | Baseline | | BCN | | Baseline | | BCN | |
| Dependent variable | CFBT_t+1_ | CFBT_t+3_ | CFBT_t+1_ | CFBT_t+3_ | EBT_t+1_ | EBT_t+3_ | EBT_t+1_ | EBT_t+3_ |
| PERF | 0.732*** | 0.570*** | 0.844*** | 0.670*** | 0.832*** | 0.577*** | 0.880*** | 0.718*** |
|  | (0.057) | (0.071) | (0.054) | (0.056) | (0.066) | (0.144) | (0.061) | (0.078) |
| NPI | 0.013 | 0.009 | 0.011 | 0.000 | -0.378*** | -0.659*** | -0.427*** | -0.729*** |
|  | (0.010) | (0.020) | (0.021) | (0.036) | (0.124) | (0.182) | (0.095) | (0.190) |
| NPI × PERF | -0.442*** | -0.448* | -0.619*** | -0.797** | 0.010 | -0.008 | -0.001 | -0.010 |
|  | (0.164) | (0.252) | (0.199) | (0.360) | (0.010) | (0.017) | (0.014) | (0.021) |
| D^ULCF^ | -0.010** | -0.004 | -0.005 | -0.003 | 0.001 | -0.003 | -0.002 | -0.010 |
|  | (0.004) | (0.007) | (0.005) | (0.007) | (0.006) | (0.009) | (0.005) | (0.008) |
| D^ULCF^ × ULCF Mean | -0.062** | -0.059** | -0.073** | -0.095*** | -0.042** | -0.095** | -0.050*** | -0.126*** |
|  | (0.028) | (0.027) | (0.030) | (0.033) | (0.021) | (0.045) | (0.017) | (0.036) |
| DTA and VD variables | YES | YES | YES | YES | YES | YES | YES | YES |
| BCN controls | NO | NO | YES | YES | NO | NO | YES | YES |
| Industry effects | YES | YES | YES | YES | YES | YES | YES | YES |
| Year effects | YES | YES | YES | YES | YES | YES | YES | YES |
| Observations | 835 | 792 | 646 | 624 | 835 | 792 | 646 | 624 |
| R^2^ | 0.647 | 0.508 | 0.653 | 0.512 | 0.623 | 0.359 | 0.647 | 0.452 |
| Adjusted R^2^ | 0.635 | 0.490 | 0.635 | 0.486 | 0.610 | 0.336 | 0.629 | 0.422 |
| The dependent variable is operating cash flow before taxes CFBT at time *t* + 1, CFBT at time *t* + 3, earnings before taxes EBT at time *t* + 1, or EBT at time *t* + 3 (in each case scaled by total assets). Estimates are calculated by OLS. Heteroscedasticity-robust standard errors are clustered at the firm level and documented in parentheses. The superscripts ***, **, and * indicate statistical significance at the 1%, 5%, and 10% levels, respectively. The term PERF is the current value of the dependent variable. Table A1 provides detailed variable definitions. ULCF Mean is the difference of the reported ULCF scaled by total assets and its sample mean. | | | | | | | | |

As documented by Table A8 to Table A10, the alternative specification of the interaction term (*D^ULCF^ x ULCF*, respectively *D^ULCF^ x ULCF Mean*) does not significantly affect its coefficient. Indeed, the coefficients of the means-adjusted interaction term in Table A10 are identical to the coefficients of the unadjusted interaction term in the baseline specification in Table A8. This underlines that that the coefficient of the interaction term captures the effect of the reported value of ULCF and not of the disclosure decition, which is captured by *D^ULCF^*. In addition, the results of Table A8 with regard to *D^ULCF^* are widely the same as for the tables A8 and A9. Again, we do not find much evidence that the disclosure of ULCF as such provides a useful indicator of future performance. The same holds for a median-adjusted version of the interaction term reported in Table A11. Here, we subtract the median of ULCF (for observations reporting this information) from the baseline value. Thus, *D^ULCF^* captures the difference between firms not reporting ULCF and firms reporting the median value of ULCF. Again, this does not have a relevant influence on our results.

**Table A11:** Median-adjusted ULCF interaction term

| Model | 1 | 2 | 3 | 4 | 5 | 6 | 7 | 8 |
| --- | --- | --- | --- | --- | --- | --- | --- | --- |
| Performance measure | Cash flow | | | | Earnings before taxes | | | |
| Reference model | Baseline | | BCN | | Baseline | | BCN | |
| Dependent variable | CFBT_t+1_ | CFBT_t+3_ | CFBT_t+1_ | CFBT_t+3_ | EBT_t+1_ | EBT_t+3_ | EBT_t+1_ | EBT_t+3_ |
| PERF | 0.732*** | 0.570*** | 0.844*** | 0.670*** | 0.832*** | 0.577*** | 0.880*** | 0.718*** |
|  | (0.057) | (0.071) | (0.054) | (0.056) | (0.066) | (0.144) | (0.061) | (0.078) |
| NPI | 0.013 | 0.009 | 0.011 | 0.000 | -0.378*** | -0.659*** | -0.427*** | -0.729*** |
|  | (0.010) | (0.020) | (0.021) | (0.036) | (0.124) | (0.182) | (0.095) | (0.190) |
| NPI × PERF | -0.442*** | -0.448* | -0.619*** | -0.797** | 0.010 | -0.008 | -0.001 | -0.010 |
|  | (0.164) | (0.252) | (0.199) | (0.360) | (0.010) | (0.017) | (0.014) | (0.021) |
| D^ULCF^ | -0.007 | -0.001 | -0.001 | 0.003 | 0.004 | 0.003 | 0.000 | -0.003 |
|  | (0.004) | (0.007) | (0.005) | (0.007) | (0.005) | (0.009) | (0.005) | (0.008) |
| D^ULCF^ × ULCF Median | -0.062** | -0.059** | -0.073** | -0.095*** | -0.042** | -0.095** | -0.050*** | -0.126*** |
|  | (0.028) | (0.027) | (0.030) | (0.033) | (0.021) | (0.045) | (0.017) | (0.036) |
| DTA and VD variables | YES | YES | YES | YES | YES | YES | YES | YES |
| BCN controls | NO | NO | YES | YES | NO | NO | YES | YES |
| Industry effects | YES | YES | YES | YES | YES | YES | YES | YES |
| Year effects | YES | YES | YES | YES | YES | YES | YES | YES |
| Observations | 835 | 792 | 646 | 624 | 835 | 792 | 646 | 624 |
| R^2^ | 0.647 | 0.508 | 0.653 | 0.512 | 0.623 | 0.359 | 0.647 | 0.452 |
| Adjusted R^2^ | 0.635 | 0.490 | 0.635 | 0.486 | 0.610 | 0.336 | 0.629 | 0.422 |
| The dependent variable is operating cash flow before taxes CFBT at time *t* + 1, CFBT at time *t* + 3, earnings before taxes EBT at time *t* + 1, or EBT at time *t* + 3 (in each case scaled by total assets). Estimates are calculated by OLS. Heteroscedasticity-robust standard errors are clustered at the firm level and documented in parentheses. The superscripts ***, **, and * indicate statistical significance at the 1%, 5%, and 10% levels, respectively. The term PERF is the current value of the dependent variable. The Appendx provides detailed variable definitions. ULCF Median is the difference of the reported ULCF scaled by total assets and its median value. | | | | | | | | |

Our additional tests suggest the following. First, we do not find evidence for a significant difference in the future firm performance of firms disclosing and not disclosing ULCF. Second, we also do not find that the dummy variable *D^ULCF^* (capturing the disclosure effect) has any relevant impact on the coefficient of the interaction term. Thus, we may conclude that the interaction term captures the effect of the disclosed value of ULCF for future firm performance while the dummy variable *D^ULCF^* captues the disclosure effect as such.

1. Similar to Joos and Plesko (2005), we also test an indicator variable for dividend-paying firms, *DIVDUM*. However, since this variable generated statistically insignificant regression results (in part with the wrong sign of the coefficient), we only report results for the specification considering the cash value of the dividend in relation to total assets. [↑](#footnote-ref-2)
2. An alternative would have been to subtract 2003 from the survey year to get a linear trend from 1 to 9. However, this would suggest an extreme and unrealistic assumption for the dynamics regarding the associations of current performance and future performance. A trend from 1 to 9 suggests that this association will change largely over a period of 9 years. Explorative analysis suggest that our trend specification resulted in more robust and realistic regression estimates regarding the association between current and future firm performance. [↑](#footnote-ref-3)
3. Indeed, as firms must be consistent in their reporting behavior (continuity in financial accounts), we expect *D^ULCF^*  to be less endogenous than *D^ULCF^ x ULCF* as the second indicator can be affected by “manipulated” by earmings management in each period in a way to either reduce or increase earnings. For example, if firms expect higher/lower future earnings, they might consider that in their accounting choices in *D^ULCF^ x ULCF.* [↑](#footnote-ref-4)
